# *Clostridium butyricum* CGMCC0313.1 Protects against Autoimmune Diabetes by Modulating Intestinal Immune Homeostasis and Inducing Pancreatic Regulatory T Cells

**DOI:** 10.3389/fimmu.2017.01345

**Published:** 2017-10-19

**Authors:** Lingling Jia, Kai Shan, Li-Long Pan, Ninghan Feng, Zhuwu Lv, Yajun Sun, Jiahong Li, Chengfei Wu, Hao Zhang, Wei Chen, Julien Diana, Jia Sun, Yong Q. Chen

**Affiliations:** ^1^Wuxi School of Medicine, Jiangnan University, Wuxi, China; ^2^State Key Laboratory of Food Science and Technology, Jiangnan University, Wuxi, China; ^3^School of Food Science and Technology, Jiangnan University, Wuxi, China; ^4^Department of Pharmacology, School of Pharmacy, Fudan University, Shanghai, China; ^5^Wuxi No. 2 Hospital, Wuxi, China; ^6^Department of Obstetrics, Nanjing Medical University Affiliated Wuxi Renmin Hospital, Wuxi, China; ^7^National Engineering Research Center for Functional Food, Jiangnan University, Wuxi, China; ^8^Beijing Innovation Centre of Food Nutrition and Human Health, Beijing Technology and Business University (BTBU), Beijing, China; ^9^Institut National de la Santé et de la Recherche Médicale (INSERM), Unité 1151, Institute Necker-Enfants Malades (INEM), Centre National de la Recherche Scienctifique, Unité 8253, Paris, France; ^10^Université Paris Descartes, Sorbonne Paris Cité, Paris, France; ^11^Department of Cancer Biology, Wake Forest School of Medicine, Winston-Salem, NC, United States

**Keywords:** type 1 diabetes, butyrate-producing bacteria, regulatory T cells, gut microbiota, regulatory T cells migration

## Abstract

Recent evidence indicates that indigenous *Clostridium* species induce colonic regulatory T cells (Tregs), and gut lymphocytes are able to migrate to pancreatic islets in an inflammatory environment. Thus, we speculate that supplementation with the well-characterized probiotics *Clostridium butyricum* CGMCC0313.1 (CB0313.1) may induce pancreatic Tregs and consequently inhibit the diabetes incidence in non-obese diabetic (NOD) mice. CB0313.1 was administered daily to female NOD mice from 3 to 45 weeks of age. The control group received an equal volume of sterile water. Fasting glucose was measured twice a week. Pyrosequencing of the gut microbiota and flow cytometry of mesenteric lymph node (MLN), pancreatic lymph node (PLN), pancreatic and splenic immune cells were performed to investigate the effect of CB0313.1 treatment. Early oral administration of CB0313.1 mitigated insulitis, delayed the onset of diabetes, and improved energy metabolic dysfunction. Protection may involve increased Tregs, rebalanced Th1/Th2/Th17 cells and changes to a less proinflammatory immunological milieu in the gut, PLN, and pancreas. An increase of α4β7^+^ (the gut homing receptor) Tregs in the PLN suggests that the mechanism may involve increased migration of gut-primed Tregs to the pancreas. Furthermore, 16S rRNA gene sequencing revealed that CB0313.1 enhanced the Firmicutes/Bacteroidetes ratio, enriched *Clostridium*-subgroups and butyrate-producing bacteria subgroups. Our results provide the basis for future clinical investigations in preventing type 1 diabetes by oral CB0313.1 administration.

## Introduction

Type 1 diabetes (T1D) is a condition in which pancreatic beta cell destruction leads to absolute insulin deficiency (1), which is caused by multiple-factors. The genetic background is essential, but not sufficient for causing the disease. T1D incidence has been rising more rapidly than can be accounted for by genetic changes, and evidence suggests that intestinal-related environmental factors, in particular the gut microbiota, are critical to the development of T1D (2, 3). The increasing incidence of T1D is most pronounced in children aged 1–5 years (4), suggesting that early life exposure is critical in shaping the autoimmune response. Moreover, young T1D patients have increased IL-4-producing cells in the small intestinal lamina propria, and interferon-γ (INF-γ)-producing cells were positively correlated with the degree of celiac disease, reflecting that gut immune reactivity is skewed in young T1D patients (5, 6). These findings raise the possibility that early life exposure to intestinal microbiota or its metabolites may be involved in immune responses associated with T1D.

Healthy children have a higher proportion of butyrate-producing bacteria in their microbiomes compared with children expressing at least one beta islet cell autoantibody (7, 8). Based on this, we speculate that the proportion of butyrate-producing bacteria may be a key regulator in determining the gut health of children at high risk of T1D. However, there is no evidence to date that any butyrate-producing bacteria protect against T1D in either animals or humans.

Additionally, several studies indicated that oral administration of CB0313.1 alleviated ovalbumin-induced allergic airway inflammation and food allergy in mice (9, 10). However, the impact of CB0313.1 on the development of T1D during different periods of life remains to be elucidated.

Recent reports have suggested that taxonomic groups Clostridia and Clostridiales prevent inflammation by a mechanism involving type 2 immunity (11), which may offer a feasible strategy to counteract T1D. Furthermore, previous studies have shown an induction of colonic regulatory T cells (Tregs) by indigenous *Clostridium* species such as *Clostridium butyricum* MIYAIRI 588 (12, 13). Thus, we speculated that supplementation with *Clostridium butyricum* CB0313.1 might induce pancreatic Tregs, thereby exerting beneficial effects on T1D.

Selective destruction of pancreatic beta cells induced by autoreactive T cells would be the primary cause of T1D. Autoimmunity is thought to escalate silently over a prolonged period of time before T1D is diagnosed; therefore, the failure to develop proper tolerogenic immune causes T1D. Immune regulation mediated by dedicated subsets of T lymphocytes, Tregs plays a critical role in immune homeostasis and self-tolerance. The major goal of immune therapies in T1D is to re-establish the balance of self-tolerance, particularly in the pancreas (14). Tregs suppress autoreactive T cells and induce immune tolerance *via* four main regulatory mechanisms: cell-to-cell contact, secretion of immuno-suppressive cytokines (e.g., TGF-β and IL-10), killing or modification of antigen-presenting cells (APCs), and competition for growth factors (15). Tregs exert their anti-diabetes effects within the pancreas as well as pancreatic lymph node (PLN) (16). Tregs in the PLN of non-obese diabetic (NOD) mice suppress the initial T cell differentiation and suppress the activation and secretion of IFN-γ, reverse the Th1/Th2/Th17 skewing to shape proper immune tolerance and prevent tissue inflammation (15–18).

Even more impressively, Tregs are easily attracted to sites of inflammation, then colocalize with and dampen the activity of Teffs. In addition, infiltration of both Teffs and Tregs was observed in the pancreas, suggesting that Tregs can be active at the inflammatory site (15).

The link between the gut and pancreas has also been emphasized in studies. Previous reports demonstrated that pancreatic islet T cells express α4β7 integrin, a gut homing receptor (6). Integrin α4β7 binds its ligands, VCAM-1 (CD106), MAdCAM-1, and fibronectin, and plays an important role in directing the migration of blood lymphocytes to the intestine and associated lymphoid tissues (19). Therefore, α4β7 can be used as a marker of gut homing cells.

Non-obese diabetic (NOD) mice share many similarities to T1D in human subjects. The incidence of spontaneous diabetes is 60–80% in the female NOD mice, whereas 20–30% in males. Therefore, we used female NOD mice in experiment (20).

Our study aimed to elucidate the effects of CB0313.1 on the progression of autoimmune diabetes and to investigate the influence of CB0313.1 on the gut microbiota, Tregs, and Th1/Th2/Th17 cell balance. This study will facilitate the development of probiotic-based therapies of T1D.

## Materials and Methods

### Animal Experiment Design

Three-week old female NOD mice (Su Pu Si Biotechnology, Co., Ltd., Suzhou, Jiangsu, China) were housed under specific pathogen-free conditions in the animal facility of Jiangnan University (Jiangsu, China). Animals had free access to water and food. All mice were housed in individual ventilated caging systems (TECNIPLAST, Italy) under SPF conditions. CB0313.1 powder and/or sodium butyrate (NaB) was suspended in sterile water, then given to the corresponding mice by gavage. Since CB0313.1 is a spore-producing probiotics, it can avoid destruction by intestinal acidity and efficiently colonize the colon (21). The animals were anesthetized, then euthanized by cervical spine dislocation. All studies were approved by the Institutional Animal Ethics Committee of Jiangnan University (JN No. 20131205) and carried out in compliance with national and international guidelines for the Care and Use of Laboratory Animals. All efforts were made to minimize animal suffering.

**Figure 1:**
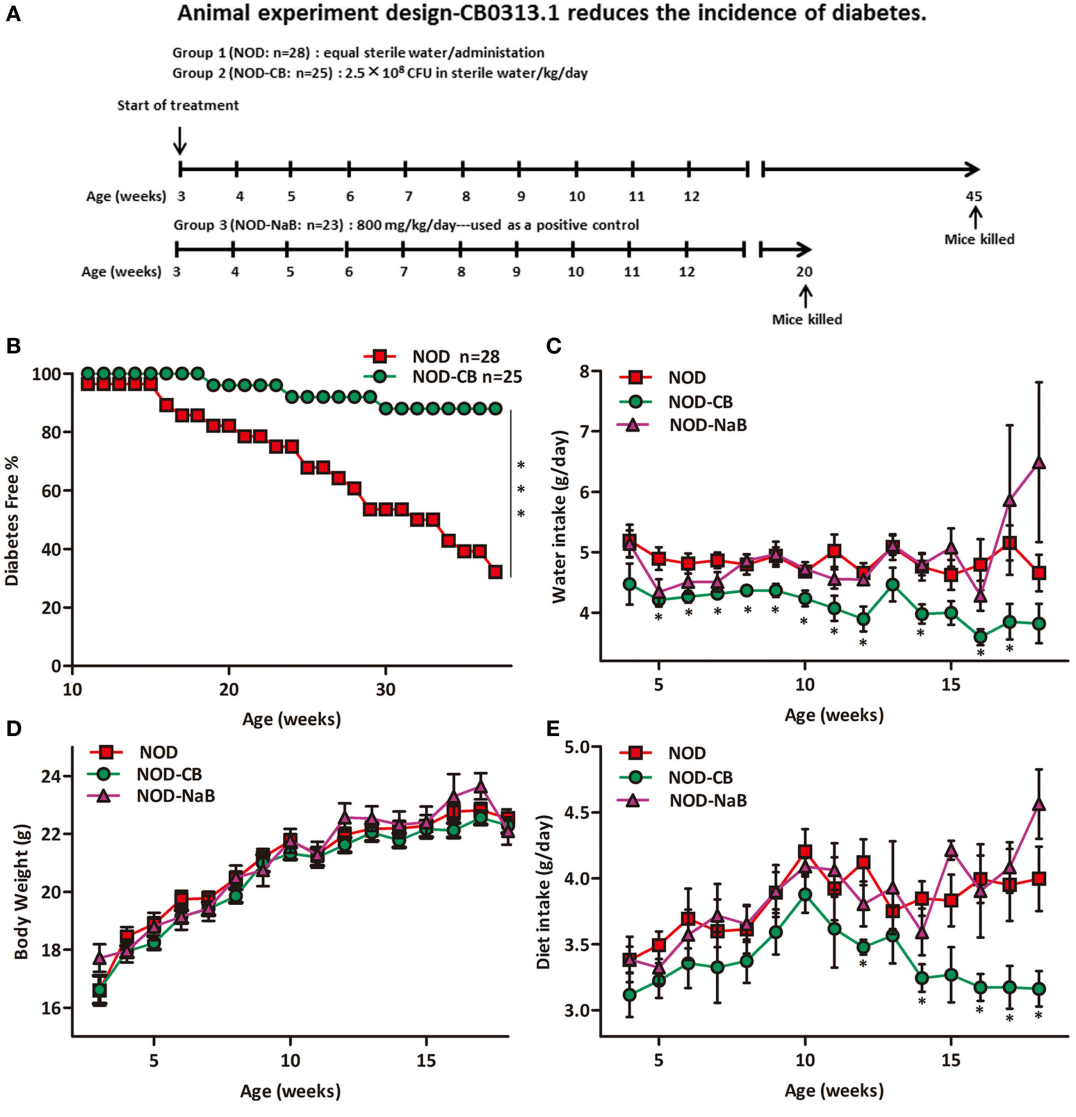

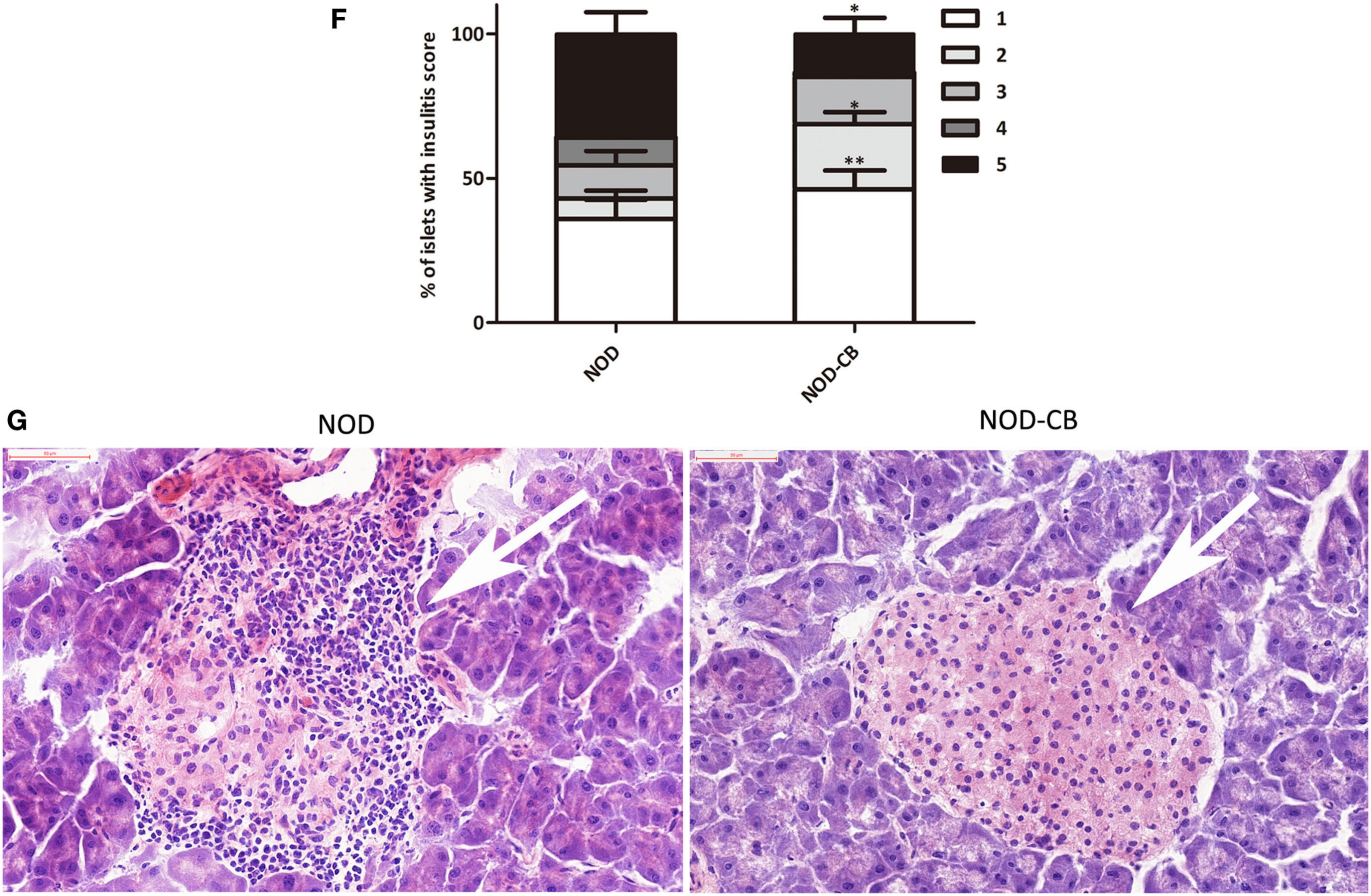
CB0313.1 delays the onset and reduces the incidence of diabetes. **(A)** To evaluate the effect of CB0313.1 on the onset of type 1 diabetes, NOD mice were randomly assigned to two groups to receive CB0313.1 or equal volumes of sterile water by gavage from 3 to 45 weeks of age. NaB was used as positive control. Mice were monitored for the appearance of clinical signs of diabetes and euthanized at disease occurrence for immune cell detection by flow cytometry. Experiment was carried up to 45 weeks of age and all remaining non-diabetic mice from all groups were euthanized for the detection of immune cells by flow cytometry. **(B)** Delayed onset and reduced incidence of diabetes in NOD-CB mice. Mice received CB0313.1, NaB, or sterile water treatment from 3 weeks of age with fasting glucose monitoring. Mice were diagnosed as diabetic and euthanized when blood glucose levels exceeded 11.1 mmol/L on 2 consecutive days along with polyuria. **(C)** Water intake. **(D)** Body weight. **(E)** Diet intake. **(F)** Insulitis score for NOD-CB and NOD control mice at 13 weeks of age. Percentage of islets with a given score at 13 weeks of age in NOD-CB (*n* = 23) and NOD control (*n* = 23) mice. 1 = white, no infiltration; 2 = light gray, few mononuclear cells infiltrated; 3 = gray, peri-insulitis; 4 = dark gray, <50% islet infiltration; 5 = black, >50% islet infiltration. **(G)** Histological examination of pancreatic islet infiltration by immune cells in female NOD mice at 13 weeks of age. Analysis of variance followed by the indicated *post hoc* test was performed to determine the significance among the three groups. *t*-test was used for two independent groups. *, **, *** *p* < 0.05, *p* < 0.01, *p* < 0.001 *vs* NOD control mice.

The study was designed as summarized in Figure 1A. To evaluate the effect of CB0313.1 administration on the onset of diabetes, the NOD mice were randomly assigned to two groups to receive CB0313.1 suspended in sterile water (2.5 × 10^8^ CFU/kg/day, 400-fold of the dosage that used in colitis patients in clinics, Qingdao East Sea Pharmaceutical Co. Ltd., Shandong, China) or equal volume of sterile water by gavage from 3 to 45 weeks of age (*n* = 25–28/group). The mice were euthanized when a diagnosis of diabetes was made or at 45 weeks of age when the study ended. At that time, all remaining mice from all groups were euthanized for immune cells detection by flow cytometry (Figure 1A).

In parallel studies, NOD-CB or NOD control mice were euthanized at 6 (*n* = 10), 9 (*n* = 9), and 13 (*n* = 23, on two independent repeats) weeks of age (after 3, 6, and 10 weeks of treatment, respectively). The MLN, PLN, pancreas, and spleen were collected for T cell detection by flow cytometry.

To block egress of lymphocytes, 9-week-old female NOD mice were either given 1.5 mg/kg FTY720 (MCE, cat. NO. HY-12005) in sterile water or equal volume of sterile water by gavage daily for a week. Next the mice were sacrificed and immune cell populations were detected in MLN, PLN, and spleen by flow cytometry.

### Blood Glucose Measurement

A glucometer (Roche, NSW, USA) was used to measure the glucose from tail vein samples, expressed in mmol/dl glucose. T1D is typically diagnosed using the criteria of the American Diabetes Association, which include acute onset of symptoms, glycosuria, plasma glucose of >11.1 mmol/l (22, 23). Polydipsia, polyphagia, and polyuria (the classic trio of symptoms associated with disease onset) along with overt hyperglycemia remain diagnostic hallmarks in children and adolescents, and to a lesser extent in adults (24, 25). In addition, NOD mice with hyperglycemia (>11.1 mmol/l) will probably require urgent implementation of insulin therapy (26, 27). Hence, NOD mice with hyperglycemia (>11.1 mmol/l) on two consecutive daily readings along with polyuria were considered to be diabetic.

### Comprehensive Laboratory Animal Monitoring System (CLAMS) Metabolic Chamber

Respiratory exchange rate (RER), spontaneous physical activity, and heat production were monitored with CLAMS (Oxymax/CLAMS system, Columbus, OH, USA). The mice (12 weeks old) were housed individually in the metabolic chambers. After 12 h of adaptation, the data for all parameters were recorded and analyzed.

### Preparation of Single Cell Suspensions

At 6, 9, 13, and 45 weeks of age, mice were sacrificed and immune cell populations were detected in mesenteric lymph nodes (MLNs), PLN, pancreas, and spleen. After the mice were euthanized, the indicated organs were placed in cold PBS immediately.

MLN and PLN were harvested and ground with frosted glass plates along with PBS scouring until there were no visible flakes, and the turbid solution was passed through a 70-µm polypropylene mesh (28).

Fresh pancreas was harvested and cut into small pieces. After being digested in PBS at 37°C for 15 min with rotation, the digested pancreatic pieces were passed through 70-µm polypropylene mesh along with grinding using a 2.5 ml syringe plunger and PBS scouring (29, 30).

Spleen was harvested and immediately ground with a 2.5 ml syringe plunger, accompanied by PBS scouring. The single spleen cell suspensions were centrifuged at 300 *g* for 5 min; then, red blood cell lysis buffer was added to lyse the red blood cells. After resting for 15 min at room temperature, the suspensions were centrifuged and resuspended in PBS (30).

### Flow Cytometry

Single cell suspensions prepared from the indicated tissues were stained for 30 min at 4°C after FcγRII/III blocking with anti-CD16/CD32 monoclonal antibody. Flow staining buffer was purchased from eBiosciences. Antibodies were purchased from eBiosciences (San Diego, CA, USA), Miltenyi (Bergisch Gladbach, Germany), and BioLegend (San Diego, CA, USA); detailed information is listed in Tables S1, S3, and S4 in Supplementary Material. For Tregs staining, cells were first surface stained, then fixed, and stained for intracellular (nuclear) forkhead box P3 (Foxp3) according to the manufacturer’s protocol. For the detection of intracellular (cytoplasmic) cytokine expression, cell suspensions were incubated at 37°C for 5 h with Cell Stimulation Cocktail (eBioscience, San Diego, CA, USA), then cells were stained and fixed according to the manufacturer’s protocol. Isotype-matched controls were included in all experiments. Stained cells were analyzed on an Attune NxT flow cytometer (Thermo Fisher Scientific, MA, USA).

### Histological Evaluation

Fresh tissues were collected at 13 weeks of age after 10 weeks of CB0313.1 administration. Tissues were fixed in NEG-50 (Thermo Scientific, MA, USA) and immediately stored at −80°C until used for frozen sections. 8-µm sections were stained with Hematoxylin and Eosin (H&E) following the standard procedure.

### Stool Sampling, DNA Extraction, and Sequencing

Stool samples were collected and immediately stored at −80°C until used for DNA extraction. Microbial genomic DNA was extracted from fecal samples using Fast DNA Spin Kit for Soil (MP Biomedicals, cat. # 6560-200, CA, USA) following the manufacturer’s instructions. The V3, V4 region of 16S rRNA was PCR-amplified using specific primers (sense: 5′-AYTGGGYDTAAAGNG-3′; antisense: 5′-TACNVGGGTATC TAATCC-3′). Reaction conditions were 95°C for 5 min; 95°C for 30 s, 64°C for 30 s, and 72°C for 30 s, then repeated for 40 cycles, with a final incubation at 72°C for 10 min. The PCR products were excised from a 1.5% agarose gel, purified by Gene Clean Turbo (MP Biomedicals, cat. #: 111102400), and quantified by Quant-iT PicoGreen dsDNA Assay Kit (Life Technologies, cat. # P7589, Carlsbad, CA, USA) following the manufacturer’s instructions. Libraries were prepared using TruSeq DNA LT Sample Preparation Kit (Illumina, cat. # FC-121-2001, San Diego, CA, USA) and sequenced for 500 + 7 cycles on Illumina MiSeq using the MiSeq Reagent Kit (500 cycles-PE, cat. # MS-102-2003). The sequences reported in this paper have been deposited in the BioProject of NCBI under the accession NO. PRJNA412689.

### Real-time PCR for Detecting Butyrate-Producing Bacteria

The final step for conversion of butyryl-CoA to butyrate is either catalyzed by butyrate kinase (*buk*) or acetate CoA-transferase (*butyryl-CoA*) kinase. Typically, these two genes are used as biomarkers for the identification/detection of butyrate-producing communities (31). Targeting the whole pathway for functional predictions is a robust way to circumvent difficulties associated with the analysis based on specific genes only (32, 33). The levels of *buk* and *butyryl-CoA* gene expression were normalized by total bacterial DNA and compared with NOD control mice. Primer sequences are given in Table S2 in Supplementary Material.

### SCFAs Analysis

Acetate, propionate, and butyrate in stool samples were analyzed by gas chromatography coupled mass spectrometry (GC-MS) (34).

### ELISA

Colonic and pancreatic TGF-β1, IL-10, IL-4, and IL-17A were measured by ELISA kit (Fcmacs, Nanjing, China) according to the manufacturer’s instructions. The tissue was homogenized with physiological saline (1:19), then the homogenate was centrifuged at 4°C for 10 min at 12,000 *g*, and the supernatant was used for ELISA analysis.

### Statistics

All data were analyzed using GraphPad Prism 5 software (San Diego, CA, USA). Cumulative diabetes incidence was calculated using the Kaplan–Meier estimation while statistical significance was evaluated by the log rank test. All data are presented as mean ± SEM (*n* = 3–28). One-way analysis of variance (ANOVA) was performed to determine the significance among three groups followed by the indicated *post hoc* test. *t*-test was used for two independent groups. *p* < 0.05 was considered statistically significant. Linear regression with a Pearson correlation analysis was performed for determining the correlation between specific bacterial clusters and fasting glucose. Optimization of 1% was performed using the unweighted pair group method with arithmetic averages clustering algorithm (UPGMA) and by principal component analysis (PCA) using Past v2.16.

## Results

### CB0313.1 Delays the Onset and Reduces the Incidence of Diabetes

The effect of CB0313.1 on the development of autoimmune diabetes in NOD mice was studied by evaluating the time of onset and the incidence of diabetes. As shown in Figure 1B, treatment at weaning (3 weeks of age) resulted in a delay of diabetes onset in NOD-CB mice (19 weeks of age) compared with the NOD control mice (11 weeks of age). In addition, at 37 weeks of age, 32.14% (9/28) of NOD control mice were diabetes free, in comparison with 88% (22/25) of mice in the NOD-CB mice (Figure 1B, *p* < 0.001 by log rank test). Moreover, CB0313.1 treatment attenuated water intake and food intake significantly, and this effect was not associated with body weight (Figures 1C–E).

Furthermore, in the group of mice treated from 3 to 13 weeks of age, we evaluated the degree of insulitis. The average insulitis score of NOD-CB mice was significantly lower than in NOD controls (Figures 1F,G; Figure S1 in Supplementary Material).

### CB0313.1 but Not Butyrate Improves Energy Metabolic Dysfunction

**Figure 2:**
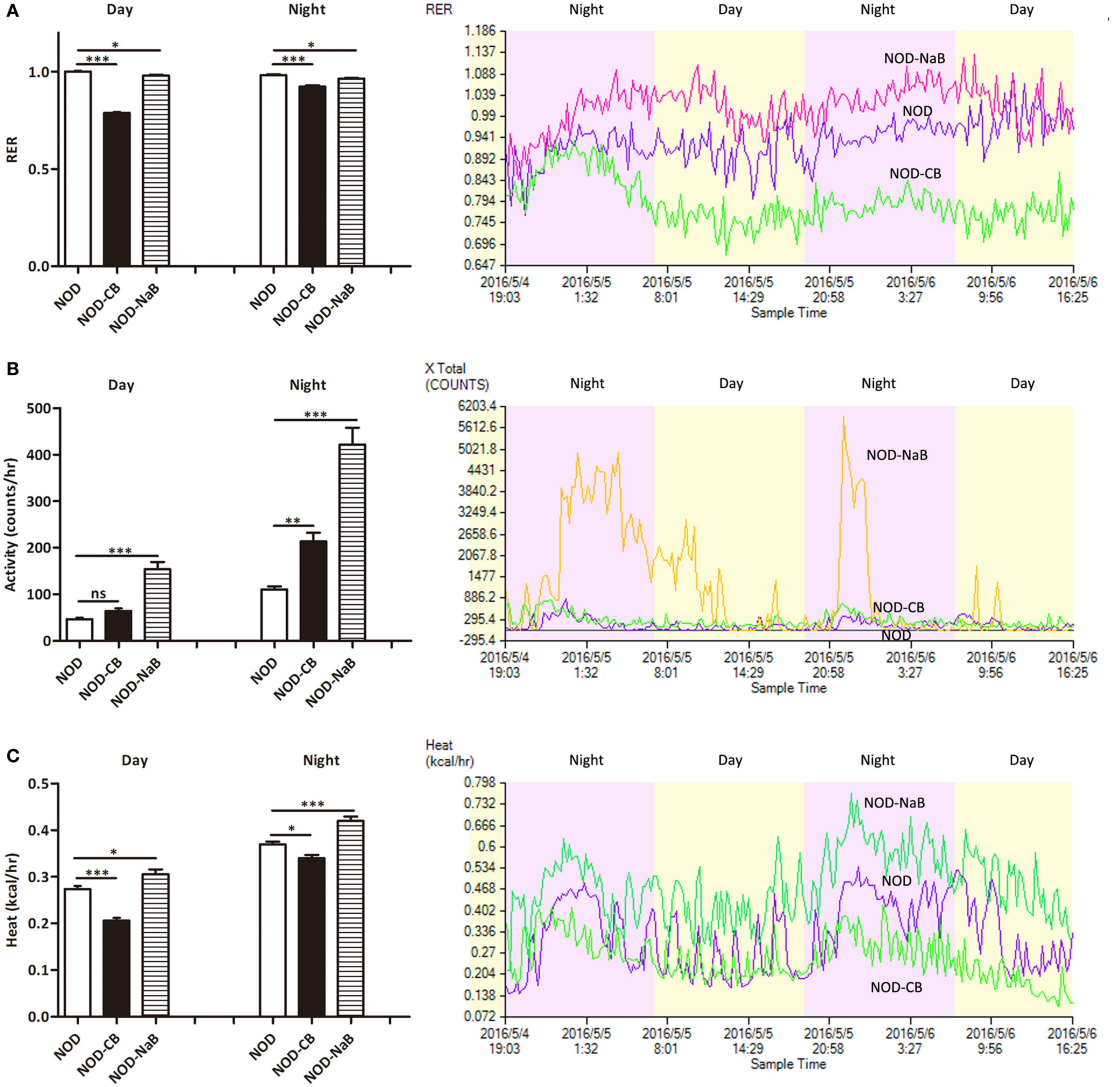
CB0313.1 but not butyrate improves energy metabolic dysfunction. Energy metabolism was examined using a metabolic chamber at 12 weeks of age. **(A)** Substrate utilization is expressed as the respiratory exchange ratio (RER), the ratio of O_2_ consumption to CO_2_ exhalation volume; **(B)** spontaneous physical activity; **(C)** heat production. Data are mean ± SEM (*n* = 4 mice per group). Analysis of variance followed by the indicated *post hoc* test was performed to determine the significance among the three groups. *t*-test was used for two independent groups. *, **, *** *p* < 0.05, *p* < 0.01, *p* < 0.001 *vs* NOD control mice.

Next, we investigated the therapeutic effect of CB0313.1 on energy metabolic dysfunction at 12 weeks of age. NOD-CB mice showed a significantly lower respiratory exchange ratio (RER) compared to NOD controls, indicating decreased glucose oxidation, and increased fat and protein oxidation in NOD-CB mice (Figure 2A, *p* < 0.001). Meanwhile, we observed significantly increased physical activity (Figure 2B, *p* < 0.01, in the nighttime) and decreased heat production (Figure 2C, *p* < 0.001 for day and *p* < 0.05 for night) in NOD-CB mice *vs* NOD controls. These findings demonstrate that CB0313.1 prevents diabetes-induced energy metabolic dysfunction, and this effect may be associated with changes in the ratio of energy expenditure, increased physical activity and decreased heat production.

To investigate how CB0313.1 affected energy metabolism, we administered NOD mice with metabolite NaB by gavage at 800 mg/kg/day from 3 weeks of age according to the recommended dosage of a previous study (35), and observed an earlier onset of T1D at 9 weeks of age in NOD-NaB mice *vs* 11 weeks of age in NOD controls (data not shown). CLAMS metabolic chambers were used to monitor metabolism of NOD-NaB mice. Interestingly, the physical activity of NOD-NaB mice was dramatically increased to 3.32-fold in the daytime, and 3.82-fold in the evening *vs* NOD controls (Figure 2B). In addition, significantly increased heat production (Figure 2C) and decreased RER (Figure 2A) were observed in NOD-NaB mice (Figure 2C). These findings demonstrate that NOD-CB mice exhibited different metabolic behaviors compared with NOD-NaB mice, indicating that the beneficial effects of CB0313.1 on T1D are associated with improvement of the energy metabolic dysfunction, but not only *via* butyrate.

### CB0313.1 Restores the Diabetes-Induced Gut Microbial Dysbiosis

**Figure 3:**
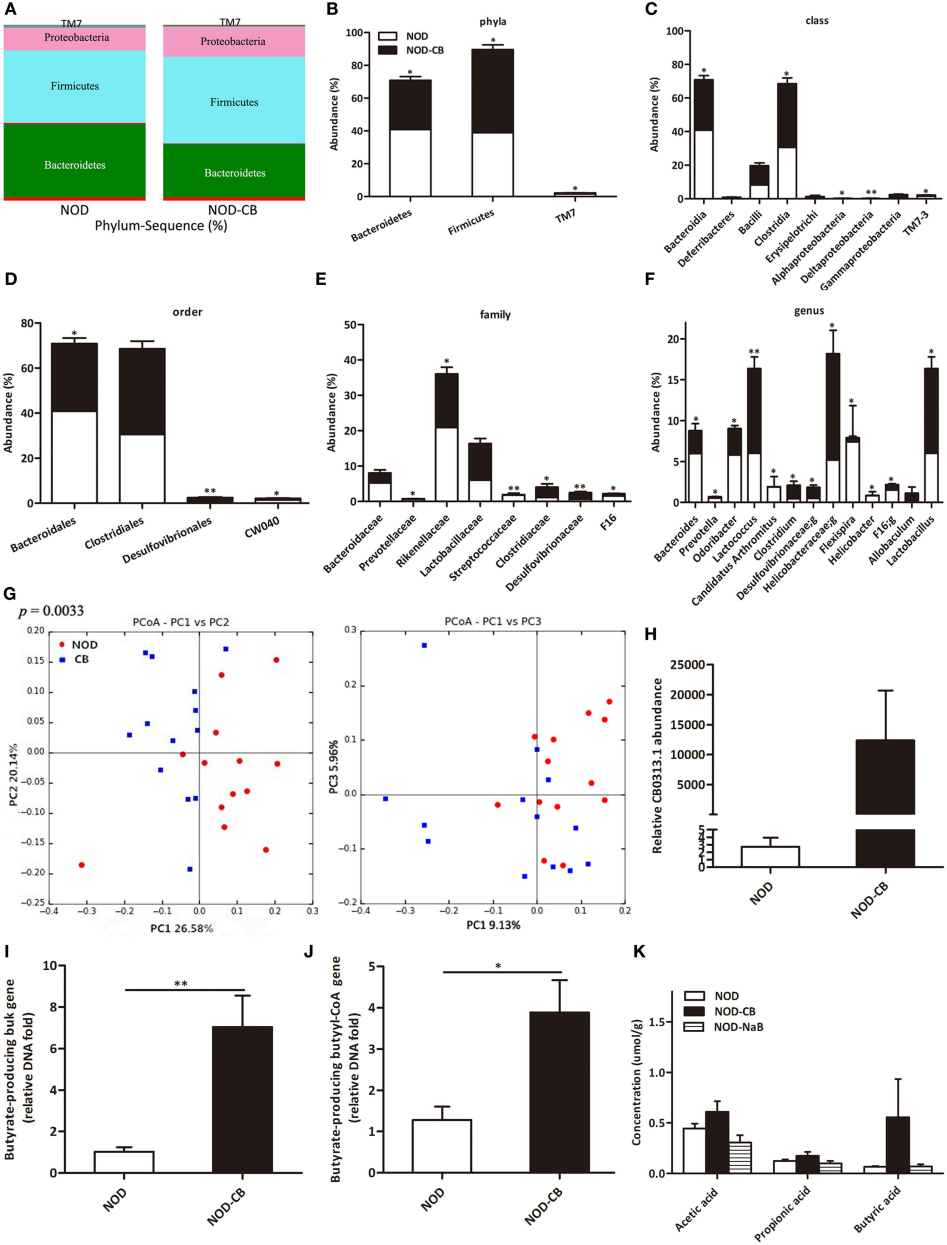
CB0313.1 restores the diabetes-induced gut microbial dysbiosis. **(A,B)** Abundance of the most important phyla in each group. Abundance of the main altered classes **(C)**, orders **(D)**, families **(E)**, and genera **(F)** in each group. **(G)** Principal coordinate analysis (PCoA) plot of weighted UniFrac distances, each dot representing a feces community; the percentage of variation explained by each principal coordinate is shown in parentheses. **(H)** Relative abundance of CB0313.1. Predominant butyrate producing genes: relative abundance of butyrate kinase (*buk*) and *butyryl-CoA* DNA in feces **(I,J)**. **(K)** SCFA concentration in feces measured by GC-MS. Analysis of variance followed by the indicated *post hoc* test was performed to determine the significance among the three groups. Data are mean ± SEM (*n* = 8 mice per group). *, **, *** *p* < 0.05, *p* < 0.01, *p* < 0.001 *vs* NOD control mice by *t*-test.

16S rRNA gene sequencing data showed that NOD-CB mice and NOD controls harbored distinct microbial communities. The vast majority (> 95%) of the annotated reads in NOD mice at 40 weeks of age were distributed among three bacterial phyla: Firmicutes, Bacteroidetes, and Proteobacteria (Figure 3A). CB0313.1 administration was associated with a significant decrease in Bacteroidetes (by 26.39%, *p* < 0.05) and a significant increase in Firmicutes (by 30.59%, *p* < 0.05) compared to NOD controls (Figures 3A,B). Figures 3C,D illustrate the microbial alterations at the class and order levels, respectively. Notably, taxonomic groups Clostridia and Clostridiales increased consistently, indicating that CB0313.1 promoted the establishment of a protective microbiome enriched in Clostridiales (38.01% in NOD-CB mice *vs* 30.56% in NOD controls) (Figures 3C,D) (11).

At the family level, 8 out of 48 families identified were markedly changed in response to CB0313.1. Major differences were observed at the level of dominant families: Bacteroidaceae, Prevotellaceae, Rikenellaceae, Streptococcaceae, and F16 decreased by 46.27%, 66.67% (*p* < 0.05), 27.56% (*p* < 0.05), 92.09% (*p* < 0.01), and 55.63% (*p* < 0.05), respectively, in NOD-CB mice *vs* NOD controls. Lactobacillaceae, Clostridiaceae, Desulfovibrionaceae increased by 49.97%, 161.26% (*p* < 0.05), 200.07% (*p* < 0.01), respectively, in NOD-CB mice *vs* NOD controls (Figure 3E).

Additionally, the following genera were increased significantly in NOD-CB mice *vs* NOD controls: *Clostridium* (by 3.54-fold, *p* < 0.05), Desulfovibrionaceae;g (by 2.76-fold, *p* < 0.05), Helicobacteraceae;g (by 2.52-fold, *p* < 0.05), *Lactobacillus* (by 1.72-fold, *p* < 0.05), and *Allobaculum* (by 111-fold), whereas the following genera decreased significantly in NOD-CB mice *vs* NOD controls: Bacteroides (by 41.5%, *p* < 0.05), *Prevotella* (by 65.43%, *p* < 0.05), *Odoribacter* (by 44.39%, *p* < 0.05), *Lactococcus* (by 93.18%, *p* < 0.01), *Candidatus Arthromitus* (by 98.94%, *p* < 0.05), *Helicobacter* (by 95.06%, *p* < 0.05), and F16;g (by 55.63%, *p* < 0.05) (Figure 3F). Most of them are still poorly characterized and could be further studied in the context of T1D.

Principal coordinate analysis (PCoA) showed that CB0313.1 significantly modified the overall structure of the gut microbiota along the first principal component (PC1) (Figure 3G, *p* < 0.01).

We found that supplementation with CB0313.1 effectively increased intestinal CB0313.1 by 12,370-fold, demonstrating its ability to efficiently colonize the gut (Figure 3H).

Typically, two genes, *buk* and *butyryl-CoA*, are used as biomarkers for the detection of butyrate-producing communities. We found that CB0313.1 only carried the *buk* gene but no *butyryl-CoA* gene, suggesting that CB0313.1 produces butyrate *via* the *buk* pathway. However, CB0313.1 administration significantly increased not only bacteria carrying the *buk* gene (by 7-fold, *p* < 0.01) but also those carrying the *butyryl-CoA* gene (by 4-fold, *p* < 0.05) (Figures 3I,J), demonstrating that probiotic CB0313.1 also promoted the growth of other butyrate-producing bacteria. These data suggest that CB0313.1 promotes the growth of intestinal butyrate-producing bacteria.

However, we observed an increase trend of butyric acid in NOD-CB mice but not in NOD-NaB mice (Figure 3K), suggesting NaB by oral gavage was not efficiently delivered to the lower digestive tract in our experiment.

Overall, the NOD-CB mice harbored more microbial clusters previously described as beneficial to T1D, whereas the NOD control mice harbored more microbial clusters previously described as pathogenic to T1D.

### Specific Bacterial Groups in the Gut Correlate with Fasting Glucose

Since the gut microbes might be involved in the gut-pancreas axis, we next investigated whether bacterial clusters from the gut microbiota were associated with fasting glucose. We performed linear regression with a Pearson correlation analysis to determine the correlation between specific bacterial clusters and fasting glucose.

**Figure 4:**
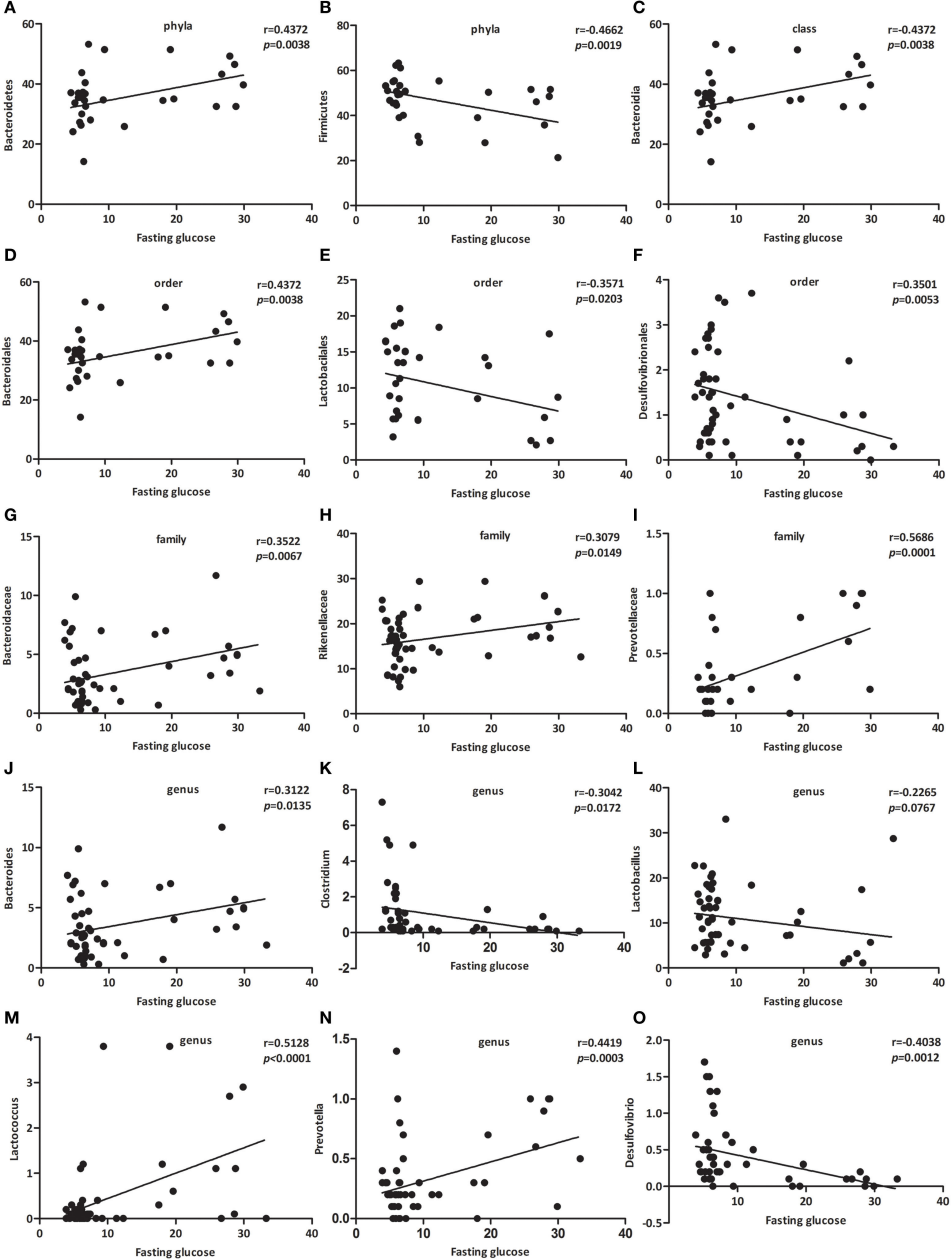
Specific bacterial groups in the gut correlate with fasting glucose. Correlation analyses between fasting glucose and the relative abundance (%) of **(A)** Bacteroidetes, **(B)** Firmicutes, **(C)** Bacteroidia, **(D)** Bacteroidales, **(E)** Lactobacilales, **(F)** Desulfovibrionales, **(G)** Bacteroidaceae, **(H)** Rikenellaceae, **(I)** Prevotellaceae, **(J)** Bacteroides, **(K)** *Clostridium*, **(L)** *Lactobacillus*, **(M)** *Lactococcus*, **(N)** *Prevotella*, **(O)** *Desulfovibrio*. *n* = 42–62. *, **, *** *p* < 0.05, *p* < 0.01, *p* < 0.001 *vs* NOD control mice by *t*-test.

As presented in Figure 4, the relative abundance of Bacteroidetes (Figure 4A, *p* < 0.01), Bacteroidia (Figure 4C, *p* < 0.01), Bacteroidales (Figure 4D, *p* < 0.05), Bacteroidaceae (Figure 4G, *p* < 0.05), Rikenellaceae (Figure 4H, *p* < 0.05), Prevotellaceae (Figure 4I, *p* < 0.001), Bacteroides (Figure 4J, *p* < 0.05), *Lactococcus* (Figure 4M, *p* < 0.001), and *Prevotella* (Figure 4N, *p* < 0.001) were positively associated with fasting glucose, whereas the relative abundance of Firmicutes (Figure 4B, *p* < 0.01), Lactobacilales (Figure 4E, *p* < 0.05), Desulfovibrionales (Figure 4F, *p* < 0.01), *Clostridium* (Figure 4K, *p* < 0.05), *Lactobacillus* (Figure 4L, *p* < 0.0767), and *Desulfovibrio* (Figure 4O, *p* < 0.01) were negatively associated with fasting glucose. We challenged these associations by performing single linear regression analyses and found that the correlations between these relative abundance and fasting glucose were significant.

Among those that were significantly correlated with fasting glucose, Prevotellaceae (belonging to the Bacteroidetes phylum) showed the most robust positive correlation (Figure 4I, *p* < 0.001). Consequently, we propose a new link between gut microbiota and fasting glucose.

### CB0313.1 Changes Cytokine Profiles and Induces Tregs in the MLN

Since the NOD-CB mice harbored a microbiome which protected against T1D onset, we hypothesized that these protective gut microbial communities might exert their beneficial effects on host intestinal immunity, which subsequently affected systemic and pancreatic immunity. To test our hypothesis, we investigated the cytokine profile of T cells from MLN.

**Figure 5:**
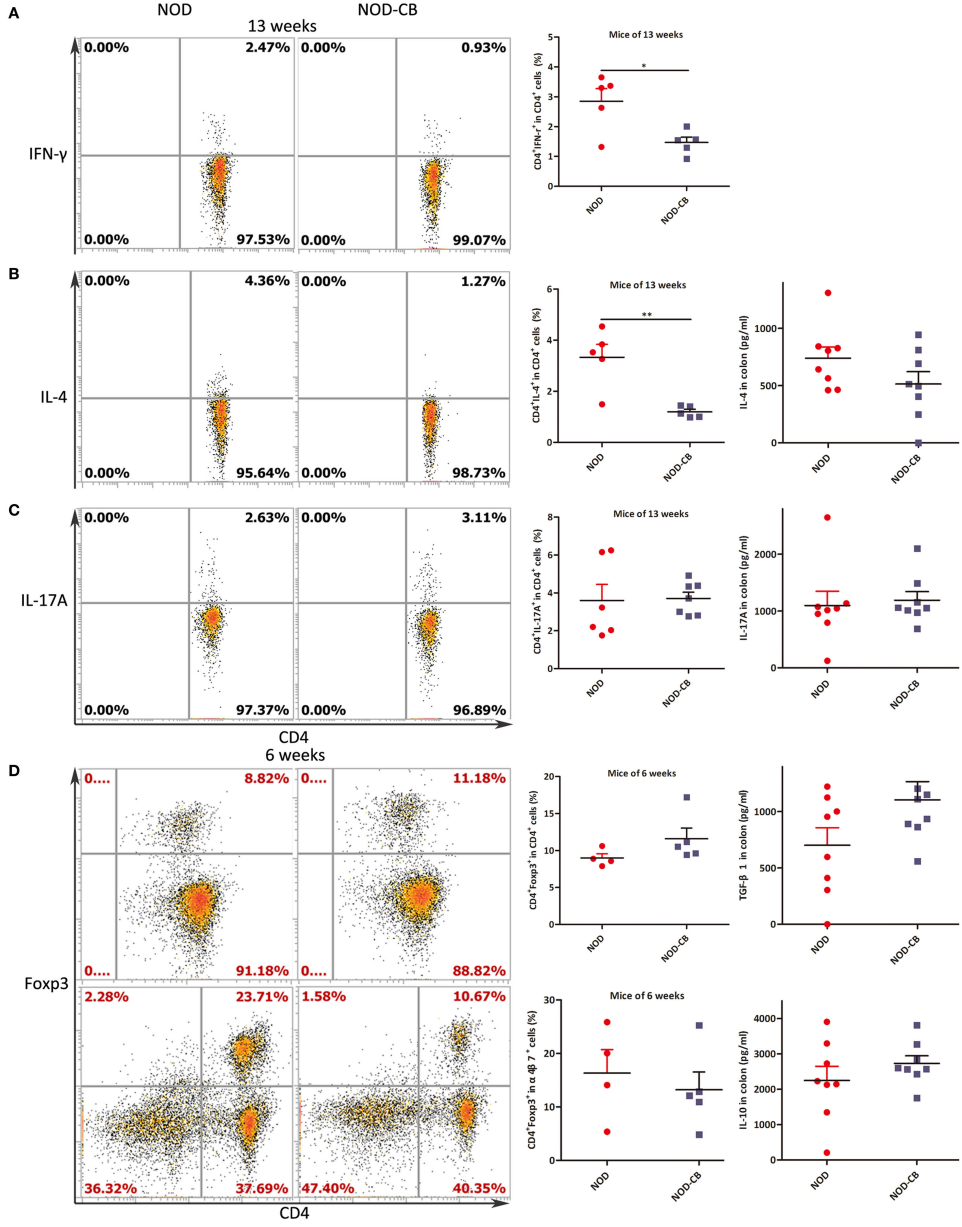

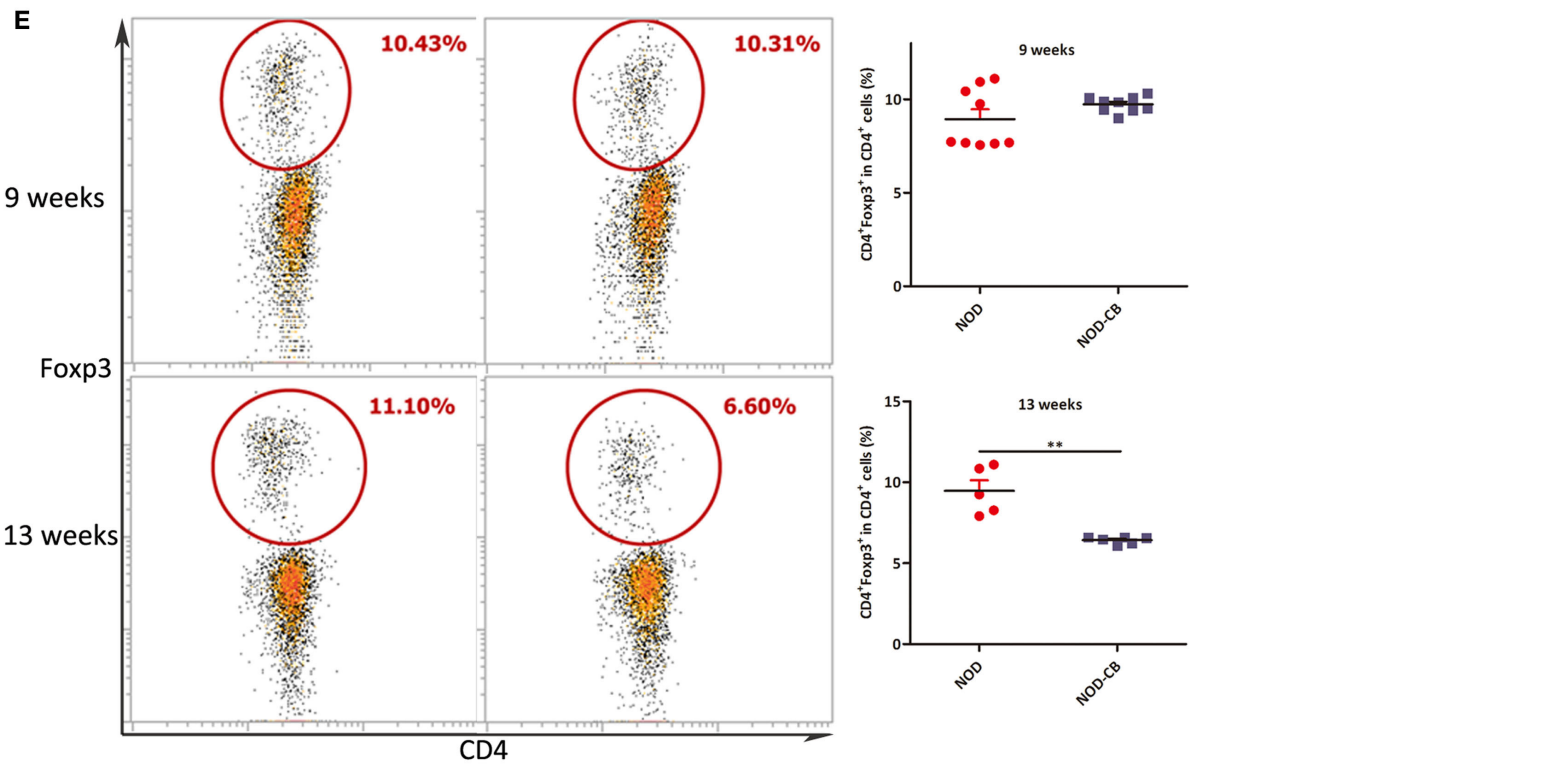
CB0313.1 changes cytokine profiles and induces Tregs in the MLN. **(A)** Percent IFN-γ^+^ in CD4^+^ cells in MLN as indicated at 13 weeks of age. **(B)** Percent IL-4^+^ in CD4^+^ cells in MLN as indicated at 13 weeks of age, and protein level of IL-4 determined by ELISA at 13 weeks of age. **(C)** Percent IL-17A^+^ in CD4^+^ cells in MLN as indicated at 13 weeks of age, and protein level of IL-17A determined by ELISA at 13 weeks of age. **(D)** Percent Foxp3^+^ in CD4^+^ cells, percent CD4^+^Foxp3^+^ in α4β7^+^ cells in MLN as indicated at 6 weeks of age, and protein levels of TGF-β, IL-10 determined by ELISA at 13 weeks of age. **(E)** Percent Foxp3^+^ in CD4^+^ cells in MLN as indicated at 9, 13 weeks of age. Data are mean ± SEM (*n* = 5–9 mice per group). *, **, *** *p* < 0.05, *p* < 0.01, *p* < 0.001 *vs* NOD control mice by *t*-test. For **(A–C,E)**, the number of cells is 5,000; For **(D)**, the number of cells is 10,000.

Intracellular staining for IFN-γ, IL-4, and IL-17A demonstrated lower percentages of IFN-γ^+^, IL-4^+^ CD4^+^ T cells and similar percentages of IL-17A^+^ CD4^+^ T cells from MLN of NOD-CB mice *vs* NOD controls at 13 weeks of age [Figure 5A (*p* < 0.05), Figure 5B (*p* < 0.01), and Figure 5C, respectively].

Intranuclear staining from MLN cells showed an increasing trend of Foxp3^+^ abundance (11.60% Foxp3^+^ in CD4^+^ T cells in NOD-CB mice *vs* 8.99% in NOD controls) at 6 weeks (Figure 5D). Among the α4β7^+^ T cells in MLN, a similar proportion of CD4^+^Foxp3^+^ cells were observed in the two groups. Moreover, at 9 weeks, a similar proportion of CD4^+^Foxp3^+^ cells were also observed in the two groups (Figure 5E).

Of note, at 13 weeks of age (when the NOD mice start to develop into T1D form insulitis phase gradually), the percentage of Foxp3^+^ in CD4^+^ T cells of NOD-CB mice markedly decrease to 6.43% (*p* < 0.001), while the percentage of NOD controls still held at 9.48% (Figure 5E). These data indicate that the Tregs in MLN of NOD-CB mice started to be reduced or migrate to other organs at 13 weeks of age.

### CB0313.1 Promotes the Migration of Gut-Primed Tregs to the PLN

**Figure 6:**
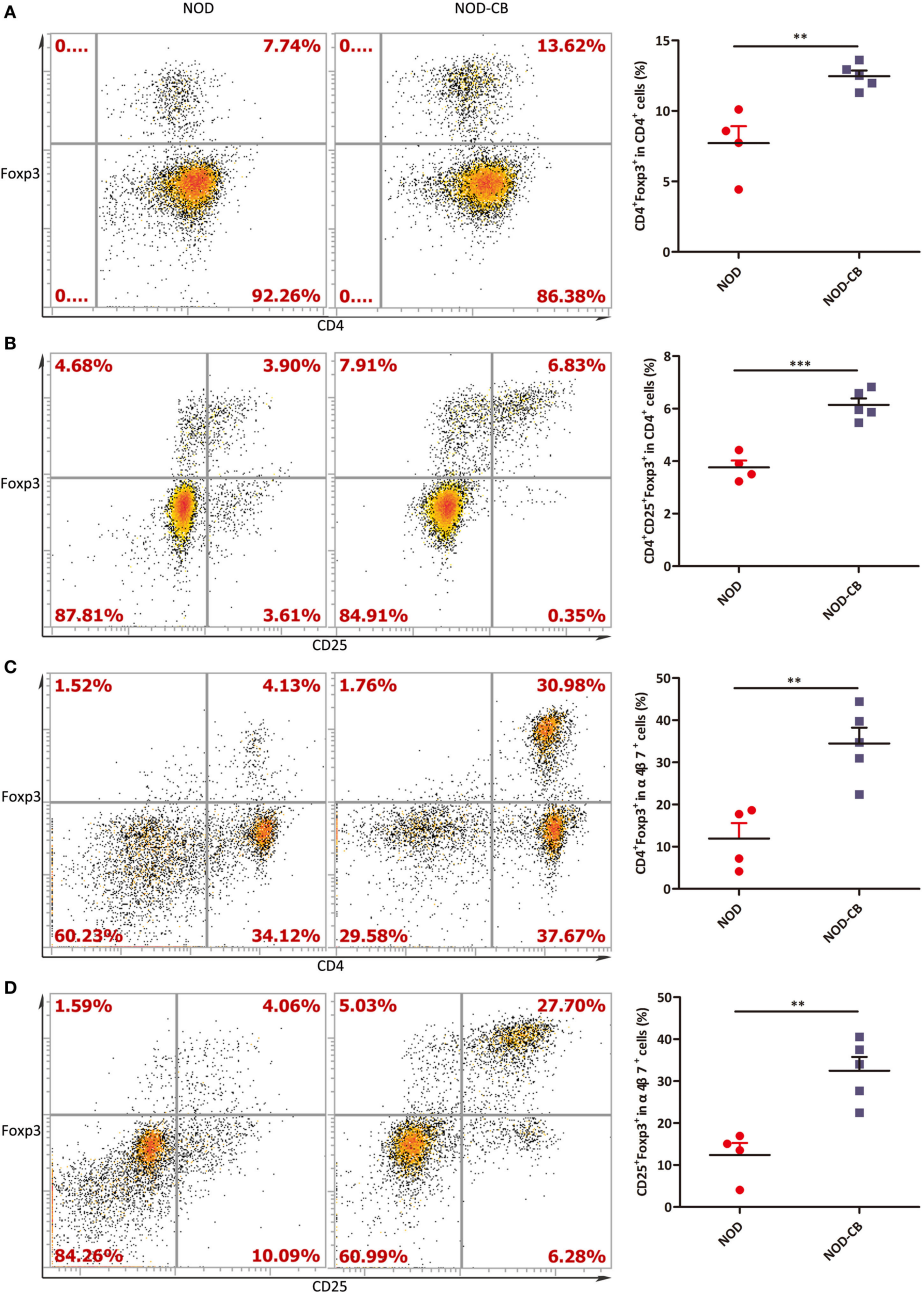
CB0313.1 promotes the migration of gut-primed Tregs to the PLN. **(A)** Percent CD4^+^ Foxp3^+^ cells in PLN. **(B)** Percent CD4^+^CD25^+^Foxp3^+^ cells in PLN. **(C)** Percent CD4^+^ Foxp3^+^ cells in α4β7^+^ cells in PLN. **(D)** Percent CD25^+^Foxp3^+^ cells in PLN. ***p < 0.05, *p* < 0.01, *p* < 0.001 *vs* NOD controls mice by *t*-test. The number of cells is 10,000.

We next examined the percentage of Foxp3^+^ in CD4^+^ T cells from PLN, and found a marked increase of Foxp3^+^ in CD4^+^ T cells in PLN (12.47% in NOD-CB mice *vs* 7.72% in NOD control mice, *p* < 0.01), as early as 6 weeks of age (Figures 6A,B). Even more impressively, the percentage of CD4^+^Foxp3^+^ in α4β7^+^ cells increased to 34.47 *vs* 11.92% in NOD controls (*p* < 0.01), suggesting that CB0313.1 promotes the migration of gut and associated lymphoid tissues-primed Tregs to the PLN (Figures 6C,D). It has been speculated that Tregs may circulate between gut and pancreas in NOD mice.

### FTY720 Suppresses the Accumulation of α4β7^+^ Tregs but Not the Total Tregs in the PLN

**Figure 7:**
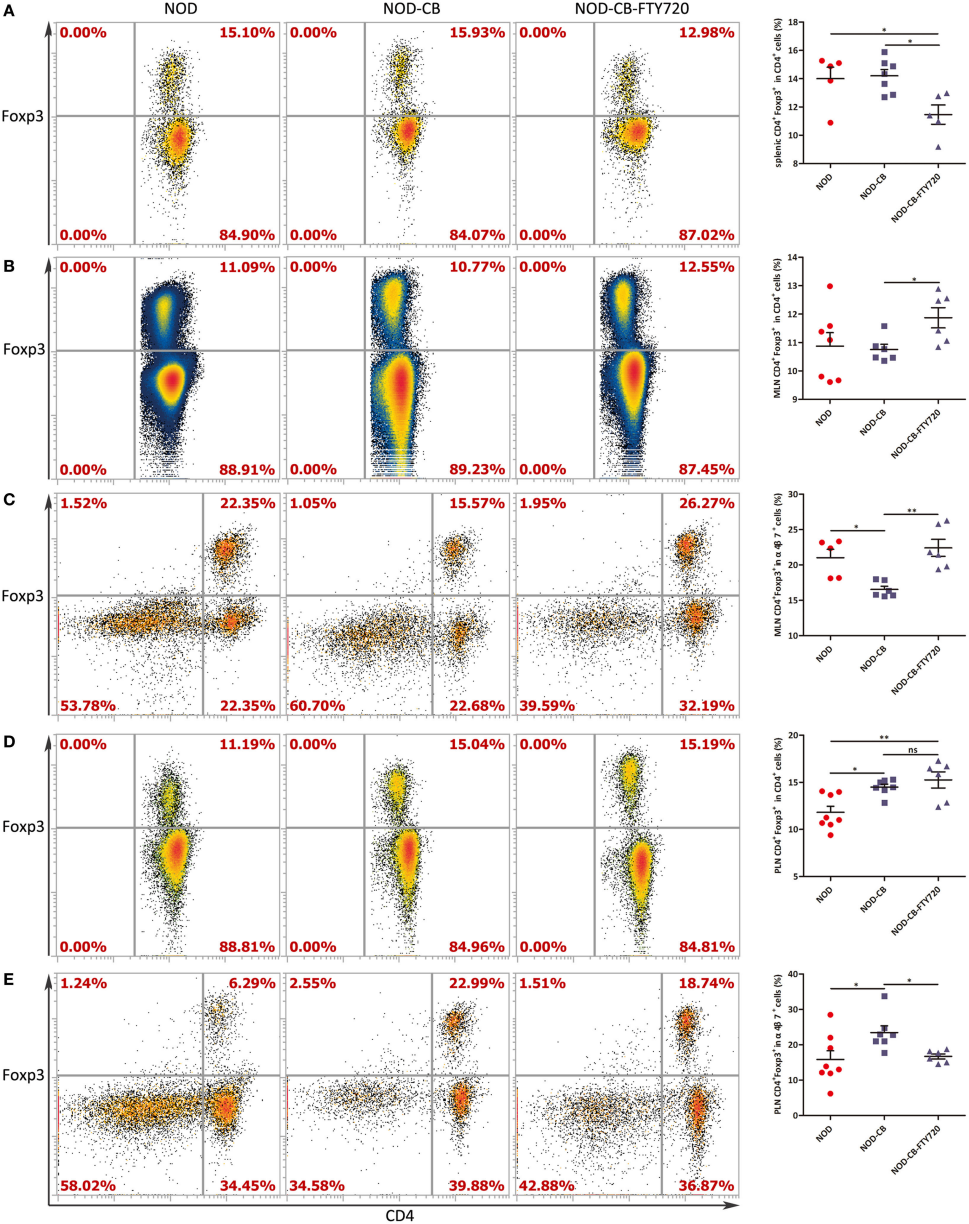
FTY720 suppresses the accumulation of α4β7^+^ Tregs but not the total Tregs in the PLN. **(A)** Percent CD4^+^Foxp3^+^ in CD4^+^ spleen cells. **(B)** Percent CD4^+^ Foxp3^+^ in CD4^+^ MLN cells. **(C)** Percent CD4^+^Foxp3^+^ in α4β7^+^ MLN cells. **(D)** Percent CD4^+^Foxp3^+^ in CD4^+^ PLN cells. **(E)** Percent CD4^+^Foxp3^+^ in α4β7^+^ PLN cells. Data are mean ± SEM (*n* = 5–8 mice per group). *, **, *** *p* < 0.05, *p* < 0.01, *p* < 0.001 *vs* NOD control mice by *t*-test. The number of cells is 10,000–300,000.

To confirm if CB0313.1 promoted the migration of gut-primed Tregs to PLN, we administrated NOD mice with FTY720 by gavage, which inhibits T cells circulation and traps them in the lymph node (36). Indeed, FTY720 resulted in a decreased proportion of splenic Tregs (Figure 7A), whereas an increased fraction of Tregs in the MLN compared to the NOD-CB mice (Figure 7B). FTY720 inhibited the migration of α4β7^+^ Tregs away from MLN (Figure 7C). In the PLN, FTY720 suppressed the accumulation of α4β7^+^ Tregs (Figure 7E), however, did not significantly change the total proportion of Tregs (Figure 7D), suggesting that the majority of Tregs in the PLN might be induced locally and migrated α4β7^+^ Tregs represented only a small fraction (Figure S6 in Supplementary Material).

### CB0313.1 Induces Pancreatic Tregs and Reduces Pancreas Inflammation

**Figure 8:**
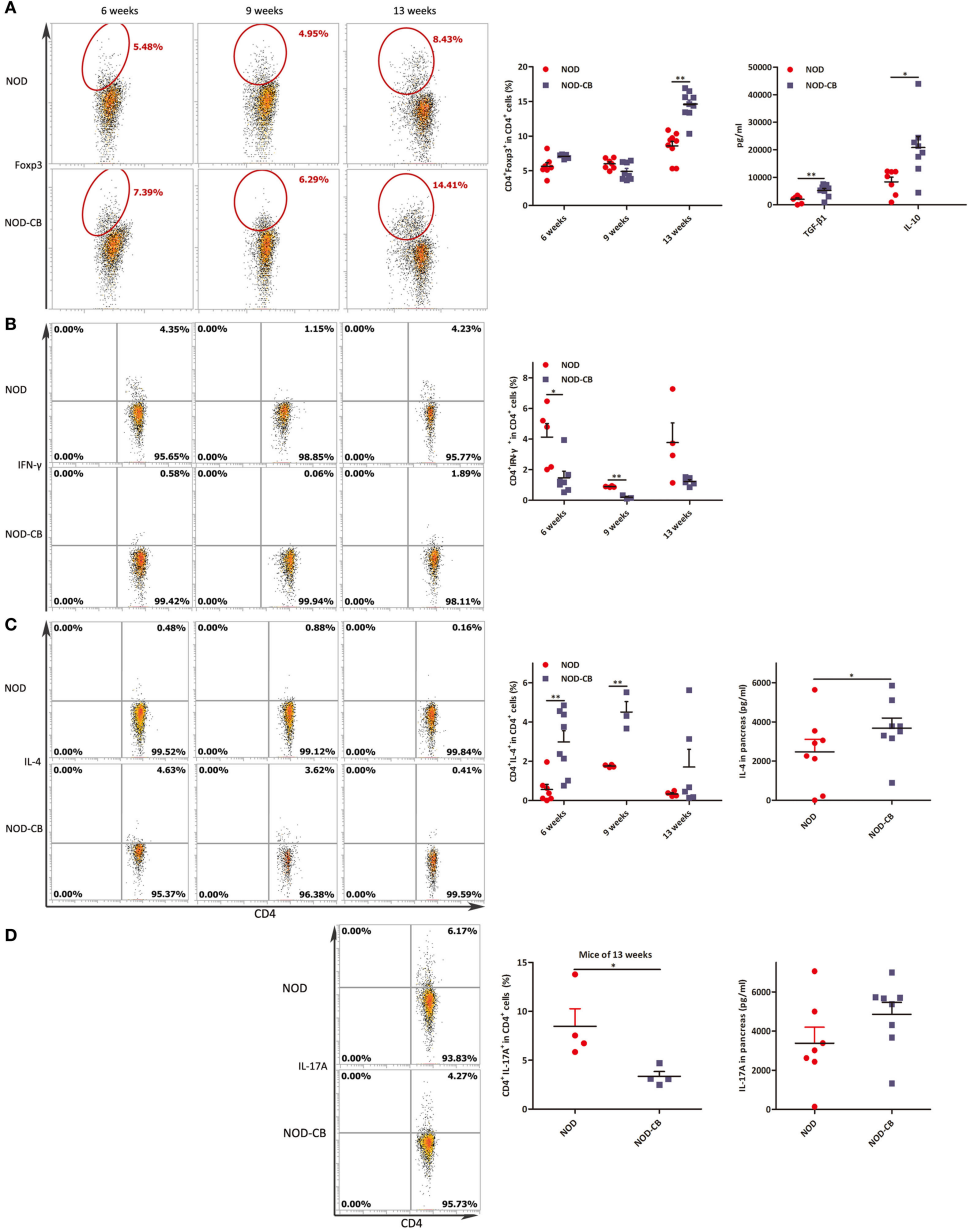
CB0313.1 induces pancreatic Tregs and reduces pancreas inflammation. **(A)** Percent CD4^+^Foxp3^+^ cells in pancreas as indicated at 6, 9, and 13 weeks of age, and protein levels of TGF-β, IL-10 determined by ELISA at 13 weeks of age. **(B)** Percent CD4^+^IFN-γ^+^ cells in pancreas as indicated at 6, 9, and 13 weeks of age. **(C)** CD4^+^IL-4^+^ cells in pancreas as indicated at 6, 9, and 13 weeks of age, and protein level of IL-4 determined by ELISA at 13 weeks of age. **(D)** CD4^+^IL-17A^+^ in pancreas as indicated at 13 weeks of age, and protein level of IL-17A determined by ELISA at 13 weeks of age. Data are mean ± SEM (*n* = 4–15 mice per group). *, **, *** *p* < 0.05, *p* < 0.01, *p* < 0.001 *vs* NOD control mice by *t*-test. For **(A)**, the number of cells is 5,000. For **(B–D)**, the number of cells is 3,000–5,000.

To elucidate the influence of CB0313.1 on pancreatic immunity, we used flow cytometry to examine the CD4^+^Foxp3^+^ T cells in the pancreas. At 6 and 9 weeks of age, we found similar proportions of pancreatic Tregs. However, at 13 weeks of age, we observed a significantly increased proportion of pancreatic Tregs in NOD-CB mice *vs* NOD controls as well as increased TGF-β and IL-10, supporting the hypothesis that oral CB0313.1 may exert its protective effects *via* Tregs and associated cytokines (Figure 8A, *p* < 0.01). Increased TGF-β in pancreas also suggests that CB0313.1 may induce Tregs *via* TGF-β related mechanism.

Although we showed that CB0313.1 could induce pancreatic Tregs at 13 weeks of age, whether this induction is a long-standing effect remained to be determined. An increased trend of pancreatic Tregs was observed in the NOD-CB mice *vs* NOD controls at the end of the experiment, indicating a higher immune tolerance locally in the pancreas due to CB0313.1 treatment (Figure S4 in Supplementary Material).

Despite the importance of Tregs in diabetes prevention, the participation of other key players cannot be ruled out. Consequently, we examined the Th1/Th2/Th17 balance in pancreas. Intracellular staining for cytokine profile of T cells from the pancreas demonstrated a lower percentage of IFN-γ^+^, IL-17A^+^ and a higher percentage of IL-4^+^ CD4^+^ T cells in NOD-CB mice *vs* NOD controls, as well as increased protein level of IL-4 (Figures 8B–D).

These findings demonstrate that CB0313.1 reduces pancreas inflammation and reversed the imbalance in Th1/Th2/Th17/Tregs.

### CB0313.1 Changes the Splenic Cytokine Profiles

**Figure 9:**
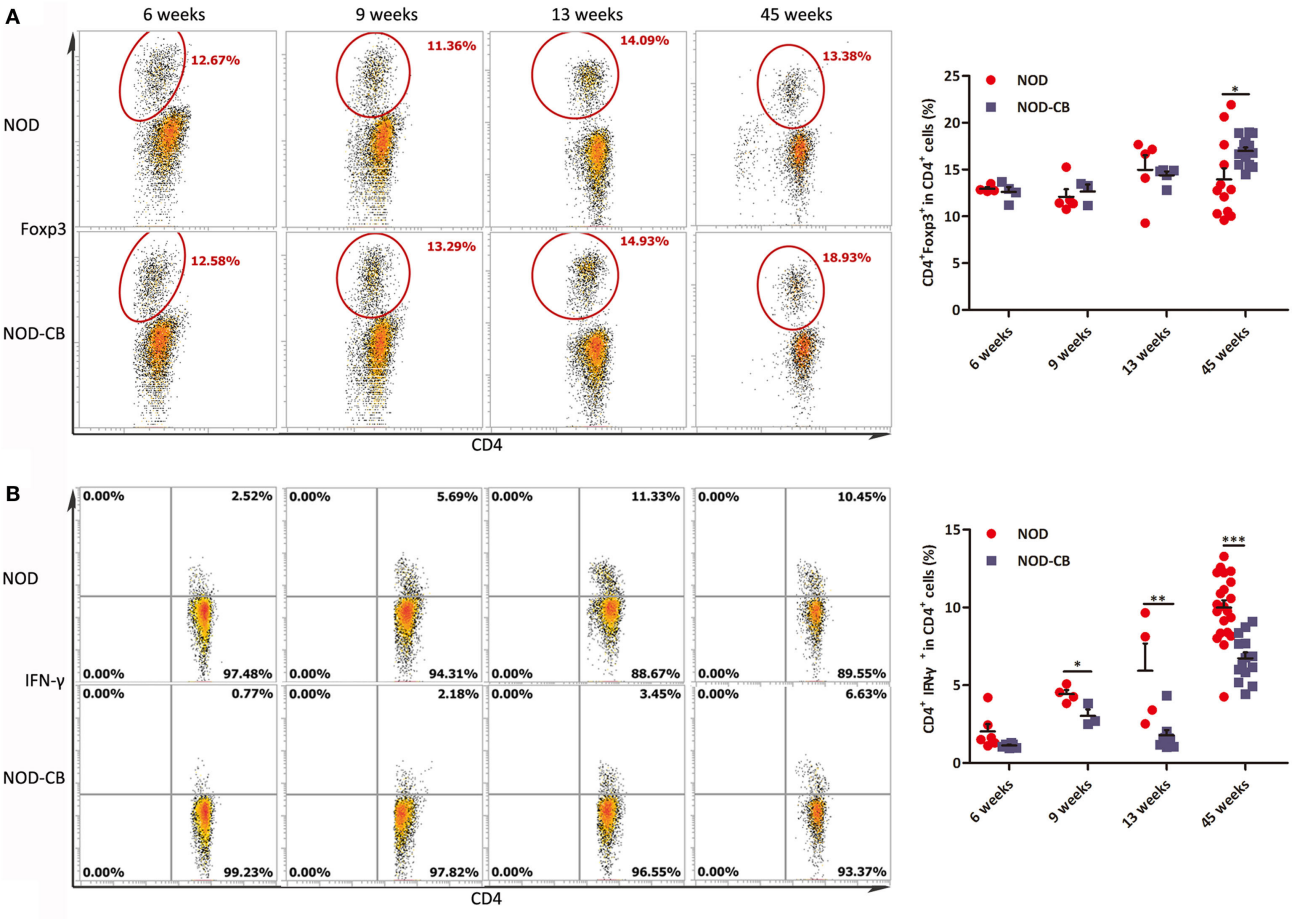

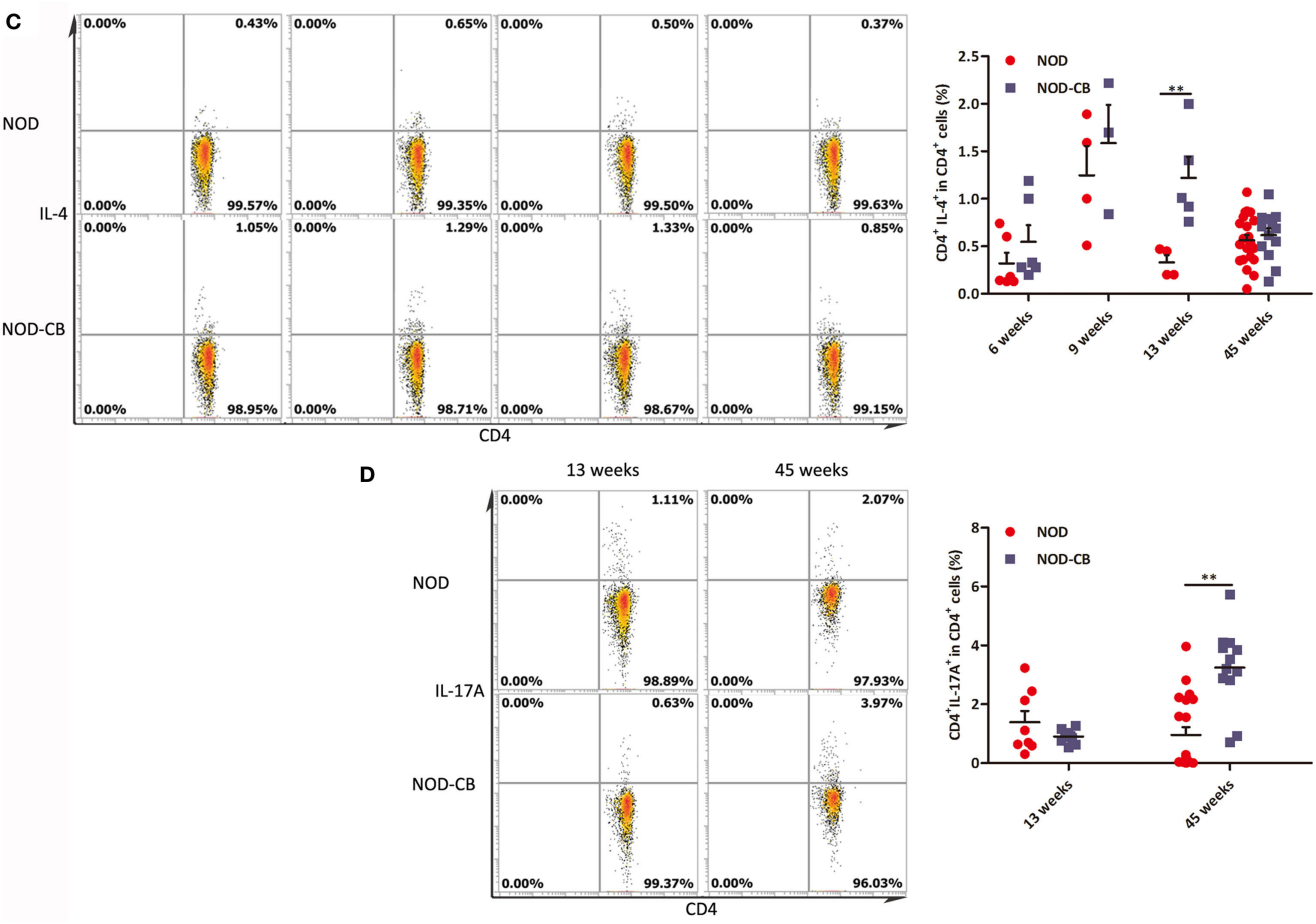
CB0313.1 changes the splenic cytokine profiles. **(A)** Percent CD4^+^Foxp3^+^ cells in spleen as indicated at 6, 9, 13, and 45 weeks of age. Percent CD4^+^IFN-γ^+^ **(B)** CD4^+^IL-4^+^ **(C)** and CD4^+^IL-17A^+^ **(D)** cells in spleen as indicated. Data are mean ± SEM (*n* = 3–21 mice per group). *, **, *** *p* < 0.05, *p* < 0.01, *p* < 0.001 *vs* NOD control mice by *t*-test. The number of cells is 10,000.

We analyzed splenic Tregs and found no differences in Treg levels among NOD-CB mice and NOD controls until 45 weeks (Figure 9A).

Furthermore, we analyzed cytokine profiles in the spleen and found a significant decrease in IFN-γ-producing Th1 cells and an increase in IL-4-producing Th2 cells (Figures 9B,C). Unexpectedly, the frequencies of Th17 cells were increased significantly in the spleen of NOD-CB mice *vs* NOD controls at 45 weeks of age (Figure 9D). These findings demonstrate that CB0313.1 decreases systemic inflammation primarily by reducing IFNγ^+^CD4^+^ T cells.

## Discussion

This study demonstrated that CB0313.1 limits the development of T1D primarily by modulation of intestinal immune homeostasis and induction of pancreatic Tregs in the early life of NOD mice. To the best of our knowledge, this is the first study that has shown a positive effect of CB0313.1 on T1D.

The development of anti-islet cell autoimmunity precedes the onset of clinical T1D and is already initiated at the age of 3–5 weeks in NOD mice (20). During the same period, dietary factors have a strong impact on the gut microbiome, which plays a central role in the development of the infant immune system (20). Therefore, weaning period (at 3 weeks of age) is an ideal time for probiotic intervention. The gut microbiome and the immune system develop synchronously. Literature shows varying effects of probiotics and antibiotics on T1D, and specific microbiome compositions may affect the risk of developing T1D (in either direction) (37). However, to date, the definitive demonstration of causal relationship regarding the specific microbiome composition and the development of T1D is still lacking (37). The main reason is that interactions between environment, gut microbiome, and the host organism were complex and numerous (38). Studies of the relationships between microbiome, immune system, and T1D may discover several microbial biomarkers of T1D, which may be used to better predict the risk for T1D. Moreover, specific bacterial or microbial metabolites can also serve as future interventions to combat autoimmune destruction of β-cells (39, 40). Our work is aimed to find out how the host immune system responds to probiotics in diabetic subjects, providing a therapeutic possibility by targeting the microbiome.

Based on the beneficial effects of CB0313.1 on preventing T1D and insulitis (Figure 1) and on improving metabolic dysfunction (Figure 2), we continued to investigate the impacts of butyrate (a major metabolite of CB0313.1) on NOD mice. However, we observed an early onset of T1D in NaB-treated mice. The dosage of NaB used in this study appeared to affect the nervous system, because the physical activity and heat production of NaB-treated mice were significantly increased *vs* NOD controls (Figure 2). As T1D patients are often accompanied by insufficient insulin, strenuous exercise may also lead to hyperglycemia and ketoacidosis (41).

Establishing causal relationship between the microbiome and physiology is critical to the ultimate goal of modifying the microbiome to prevent or treat diabetes. A key for deriving a mechanistic explanation of the described hypotheses may lie in the metabonomics analysis of intestinal contents from CB0313.1-treated mice.

We found that CB0313.1 treatment could increase the levels of acetic acid, propionic acid, and butyric acid in high fat diet mice model in our previous study; however, in this study, we only observed an increased trend of butyric acid in NOD-CB mice (Figure 3K). We did not treat our mice with acetate or propionate alone. There are several reports about propionic acid improving glycometabolism *via* several mechanisms, such as GLP-1, FFAR2, FFAR3, and intestinal gluconeogenesis, etc. (42). Therefore, it is possible that propionic acid can protect against T1D; however, there is no detailed investigation on the effect of propionic acid on T1D to date.

We found that Bacteroidetes abundance was significantly decreased in NOD-CB mice *vs* NOD controls (Figures 3A,B). The relative abundance of Bacteroidetes (Figure 4A), Bacteroidia (Figure 4C), Bacteroidales (Figure 4D), Bacteroidaceae (Figure 4G), and Bacteroides (Figure 4J) were positively associated with fasting glucose. Furthermore, the relative abundance of Rikenellaceae, Prevotellaceae, Prevotella (belonging to the Bacteroidetes phylum) also showed positive correlation with fasting glucose in our study (Figures 4H,I,N). This is consistent with previous studies showing that the Bacteroides subgroups positively correlated with increased risk of early autoantibody development and decreased butyrate-producing bacteria (43, 44).

Clostridia is one of the most prominent Gram-positive and spore-forming bacterium indigenous to the murine gastrointestinal tract; and it becomes prominent after weaning and persists in the adult animals (45). Moreover, some *Clostridium* clusters were shown to induce colonic Tregs (13) and were involved in the maintenance of mucosal homeostasis (11) as well as the prevention of inflammatory bowel disease (46, 47). In our study, taxonomic groups Clostridia, Clostridiales, Clostridiaceae, and *Clostridium* were increased consistently in NOD-CB mice *vs* NOD controls (Figures 3B–F), and we found that the relative abundance of *Clostridium* was negatively associated with fasting glucose (Figure 4K), indicating that CB0313.1 formed an environment with enriched *Clostridium*-clusters. Thus, we speculate that CB0313.1 might be involved in type 2 immunity, inducing Tregs, thereby retarding and/or suppressing the onset of T1D (48, 49).

Although CB0313.1 only carries the *buk* gene, oral administration of CB0313.1 also causes a significant increase in butyrate-producing bacteria carrying the *butyryl-CoA* gene (Figure 3J), which might be explained by a modification of the other butyrate-producing bacteria by CB0313.1 *via* an unknown mechanism. However, this hypothesis merits further investigation.

Thus, the selective modulation of gut microbial phenotypes, particularly the enrichment of *Clostridium* subgroups and of the butyrate-producing bacteria, may contribute to the improvement of T1D and associated immune imbalance. Therefore, we speculate that CB0313.1 may exert its beneficial effect via itself and/or through its modification of the gut microbiota composition.

Previous reports have demonstrated that CB0313.1 alleviated intestinal inflammation (50), but the exact mechanism remains elusive. Intracellular staining for cytokine profiles of T cells from MLN demonstrated lower percentages of IFN-γ^+^, IL-4^+^ and similar percentages of IL-17A^+^CD4^+^ T cells in NOD-CB mice *vs* NOD controls (Figure 5). These findings are consistent with previous observations of young T1D patients (6).

Two weeks after birth, the peripheral lymph nodes of mice start to separate into T zone and B zones, whereas in germ-free mice, the development is repressed, suggesting that the gut microbiota affects the development of peripheral lymph nodes (51). In addition, recent reports indicated that the frequency of Foxp3^+^ Tregs in colonic and small intestinal lamina propria increased after weaning (13), indicating that dietary factors influence the accumulation of intestinal Tregs, particularly after weaning. In our study, at 6 weeks of age, the percentages of CD4^+^ T cells in MLN and PLN were significantly greater in NOD-CB mice *vs* NOD controls (Figure S5 in Supplementary Material), indicating that CB0313.1 promotes the development of MLN and PLN after weaning, but the molecular mechanism is unknown.

Recent reports have suggested that gut-associated lymphoid tissues might play a critical role in islet-specific autoimmunity in diabetes-prone individuals, even in humans (52–55). Consistent with these studies, we observed a significantly decreased MLN Tregs at 13 weeks of age accompanied by an increased PLN and pancreatic Tregs at in NOD-CB mice (Figures 5E and 6A). Consequently, we speculated that the MLN Tregs might migrate to PLN *via* blood circulation or lymphocinesia in response to CB0313.1. Therefore, we used α4β7 as a gut homing marker to detect the trend of MLN Tregs. At 6 weeks of age, in the PLN, the percentage of CD4^+^Foxp3^+^ among α4β7^+^ cells increased markedly, suggesting that CB0313.1 promotes the migration of gut-primed Tregs to the PLN. However, FTY720 experiment indicated that the majority of Tregs in the PLN might be induced locally and migrated α4β7^+^ Tregs represented only a small fraction (Figure 7).

In addition, we found CB0313.1 treatment can effectively elevate the frequency of splenic CD11c^+^ in CD45^+^ cells (Figure S7 in Supplementary Material). Furthermore, an increased pancreas TGF-β was observed. Taken together, we speculate that in the pancreas and PLN, the Tregs may be induced by the DCs, TGF-β, or some other way.

It seems that CB0313.1 contributes to the reduced onset of diabetes by the gut-pancreas axis. However, the exact mechanism of immune cell circulation between gut and pancreas needs to be further investigated.

Moreover, in the PLN and pancreas, a higher proportion of CD4^+^ T cells was also marked with Foxp3^+^ in NOD-CB mice (Figures 6A, 7D and 8A), indicating a higher immune tolerance environment.

IL-17A is generally considered to be a proinflammatory cytokine in T1D (56). However, we analyzed splenic cytokine profiles and found the splenic IL-17A level significantly increased at the end of study in response to CB0313.1 (Figure 9D). It is possible that promoting insulitis, pancreatic inflammation (57), and progression to T1D (58, 59) only occurs after the conversion of Th17 cells to Th1, while in our study the IFNγ-producing Th1 cells did not increase.

**Figure 10:**
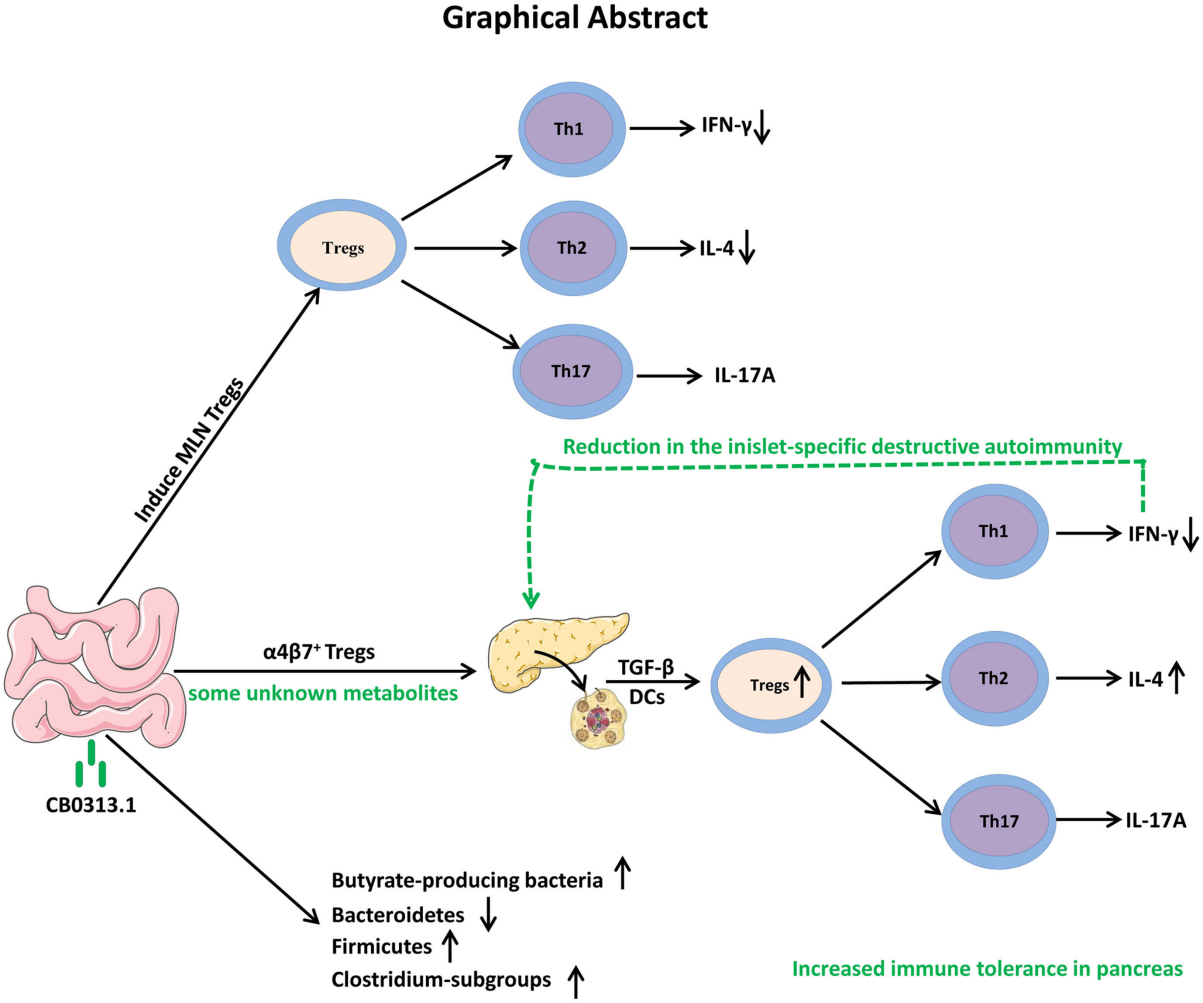
Graphical abstract. Early oral CB0313.1 administration (starting at weaning in diabetes-prone NOD mice) induced Tregs in PLN and pancreas, balanced pancreatic Th1/Th2/Th17 cells, enhanced Firmicutes/Bacteroidetes, enriched *Clostridium*-subgroups and butyrate-producing bacteria subgroups, eventually preventing the onset and progression of type 1 diabetes.

In conclusion, our study demonstrates that oral CB0313.1 administration (starting at weaning) in diabetes-prone NOD mice induces the accumulation of PLN and pancreatic Tregs, which is associated with a reduction in the islet infiltrates and islet-specific destructive autoimmunity, eventually preventing the onset and progression of diabetes (Figure 10). Given the absence of side effects of CB0313.1 treatment for 45 weeks in NOD mice and the demonstrated safety of CB0313.1 in patients with gut related diseases (21, 50), CB0313.1 is likely to be a relative safe agent. Our results provide a rationale for future clinical trials on primary prevention of T1D by oral CB0313.1 administration.

## Ethics Statement

All studies were approved by the Institutional Animal Ethics Committee of Jiangnan University (JN. No 20131205) and carried out in compliance with national and international guidelines for the Care and Use of Laboratory Animals.

## Author Contributions

YC, JS conceived the idea and reviewed the final manuscript. YC, JS, LJ designed the experiments. LJ wrote the manuscript with the assistance of YC and JS. LJ, KS, NF, YS, and JL performed flow cytometry. LJ performed the other experiments. LJ, L-LP, and CW analyzed the data. L-LP, ZL, HZ, WC, and JD revised the manuscript, provided intellectual input, and contributed to data acquisition. All authors participated in the discussion and commented on the paper.

## Conflict of Interest Statement

The authors declare that the research was conducted in the absence of any commercial or financial relationships that could be construed as a potential conflict of interest.

## Supplementary Material

Figure S1 Insulitis score for non-obese diabetic (NOD)-CB and NOD control mice. Percentage of islets with a given score at 13 weeks of age in NOD-CB (*n* = 23) and NOD control (*n* = 23) mice. **(A)** 1 = white, no infiltration; **(B)** 2 = light gray, few mononuclear cells infiltrated; **(C)** 3 = gray, peri-insulitis; **(D)** 4 = dark gray, <50% islet infiltration; **(E)** 5 = black, >50% islet infiltration. Data are mean ± SEM. **p* < 0.05, ***p* < 0.01.

Figure S2 **(A)** Relative clostridial cluster IV abundance; **(B)** Relative clostridial cluster XIVa abundance. Data are mean ± SEM, (*n* = 3–5 mice per group). *, **, *** *p* < 0.05, *p* < 0.01, *p* < 0.001 *vs* NOD control mice by *t*-test.

Figure S3 Methods of gating for FACS.

Figure S4 Percent CD4^+^Foxp3^+^ cells in pancreas as indicated at 45 weeks of age.

Figure S5 Percent CD4^+^ cells in MLN and PLN. **(A)** Percent CD4^+^ cells in MLN as indicated at 6, 9, and 13 weeks of age in the previous experiments using the antibody of CD4 (eBioscience, FITC). **(B)** Percent CD4^+^ cells in MLN e as indicated at 6 weeks of age in the additional experiments using the new antibody of CD4 (Miltenyi, PE-vio770). **(C)** Percent CD4^+^ cells in PLN as indicated at 6 weeks of age in the additional experiments using the antibody of CD4 (Miltenyi, PE-vio770).

Figure S6 Percent α4β7^+^ cells in PLN Tregs. Data are mean ± SEM (*n* = 5–8 mice per group). *, **, *** *p* < 0.05, *p* < 0.01, *p* < 0.001 *vs* NOD control mice by *t*-test.

Figure S7 Percent CD11c^+^ cells in splenic CD45^+^ cells. *, **, *** *p* < 0.05, *p* < 0.01, *p* < 0.001 *vs* NOD control mice by *t*-test.

Table S1 Information of antibody for FACS.

Table S2 Primer sequences used in this study for microbial abundance.

Table S3 Information of antibody for FACS for the experiment of regulatory T cells migration.

Table S4 Information of antibody for FACS for the experiments of CD45^+^CD11c^+^.
