# Supplementary figures and images for: Clostridium butyricum CGMCC0313.1 Protects against Autoimmune Diabetes by Modulating Intestinal Immune Homeostasis and Inducing Pancreatic Regulatory T Cells
Source: Front Immunol. 2017 Oct 19;8:1345. doi: 10.3389/fimmu.2017.01345 (PMC5654235; doi:10.3389/fimmu.2017.01345)

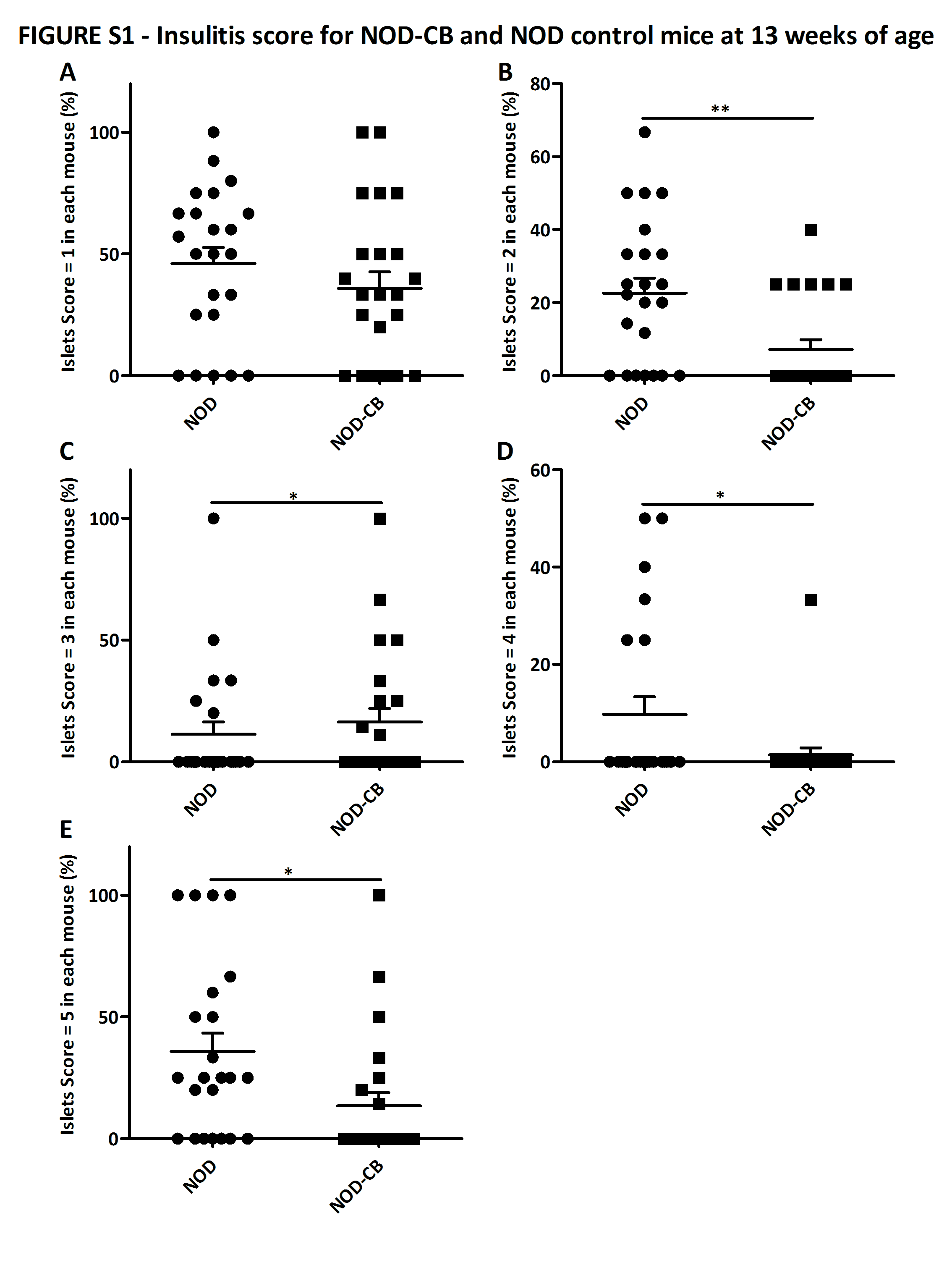

Supplement: Figure S1 — Insulitis score for non-obese diabetic (NOD)-CB and NOD control mice. Percentage of islets with a given score at 13 weeks of age in NOD-CB (n = 23) and NOD control (n = 23) mice. (A) 1 = white, no infiltration; (B) 2 = light gray, few mononuclear cells infiltrated; (C) 3 = gray, peri-insulitis; (D) 4 = dark gray, <50% islet infiltration; (E) 5 = black, >50% islet infiltration. Data are mean ± SEM. *p < 0.05, **p < 0.01. [file image_1.tif]

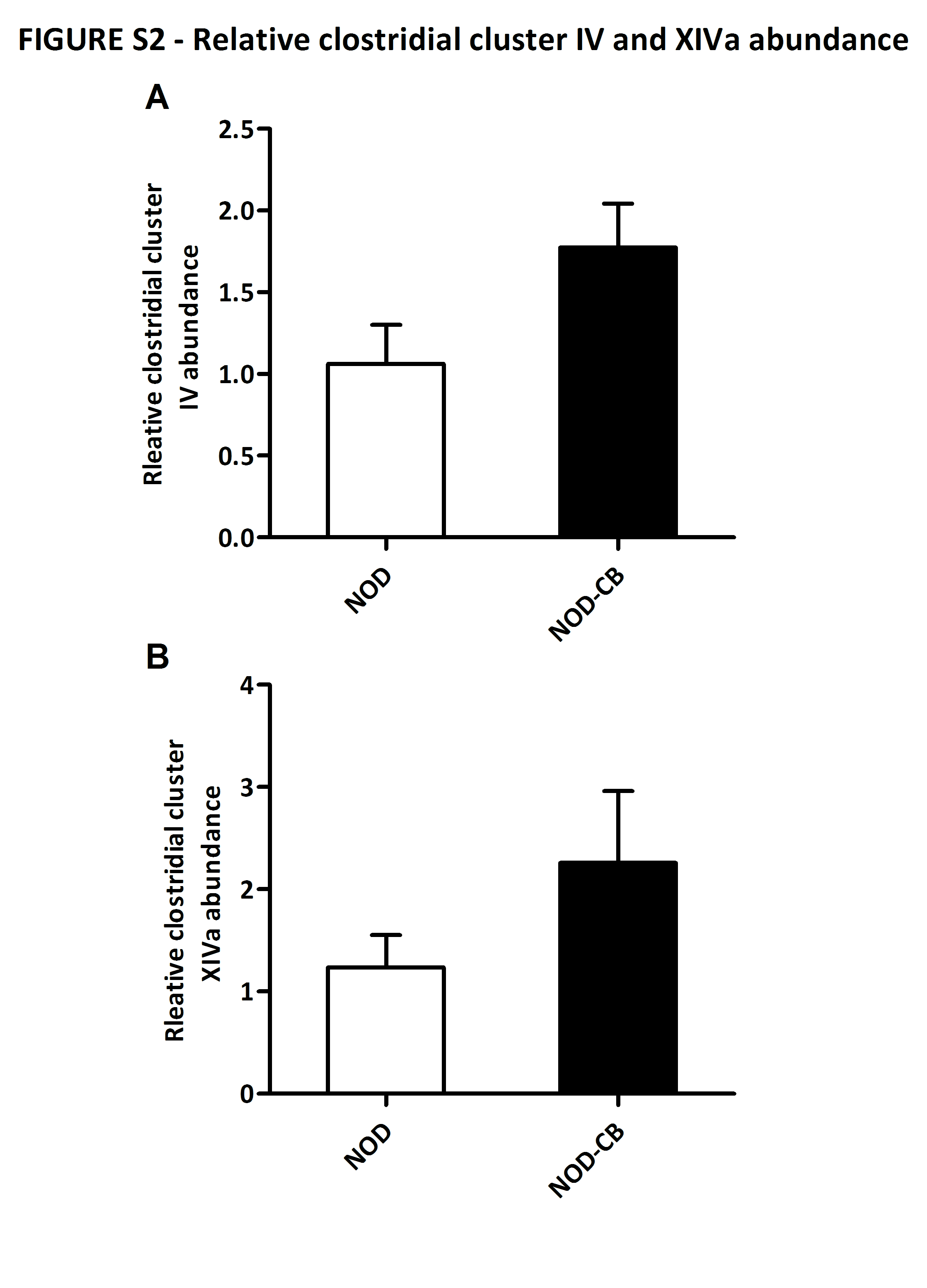

Supplement: Figure S2 — (A) Relative clostridial cluster IV abundance; (B) Relative clostridial cluster XIVa abundance. Data are mean ± SEM, (n = 3–5 mice per group). *, **, *** p < 0.05, p < 0.01, p < 0.001 vs NOD control mice by t-test. [file image_2.tif]

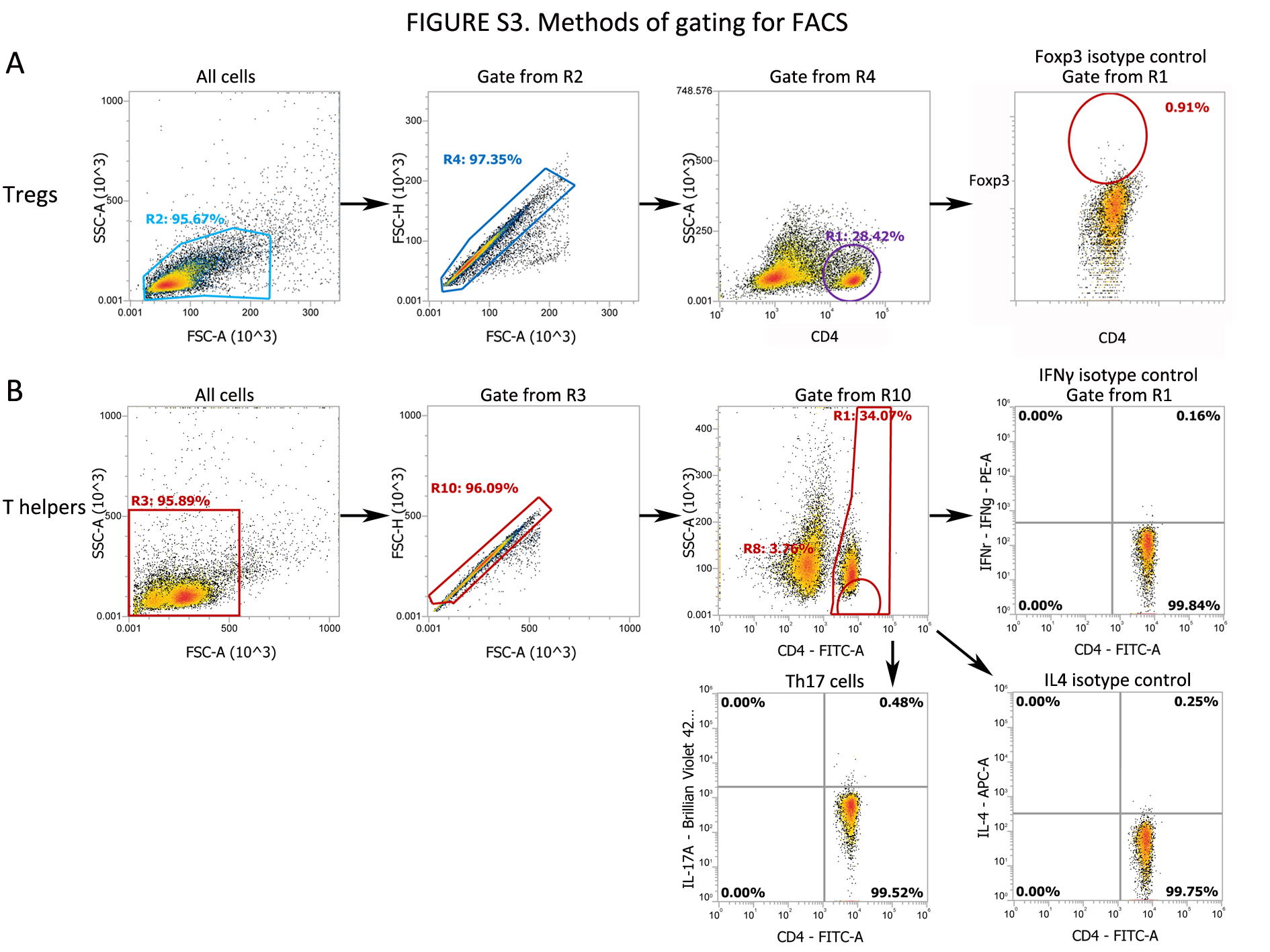

Supplement: Figure S3 — Methods of gating for FACS. [file image_3.tif]

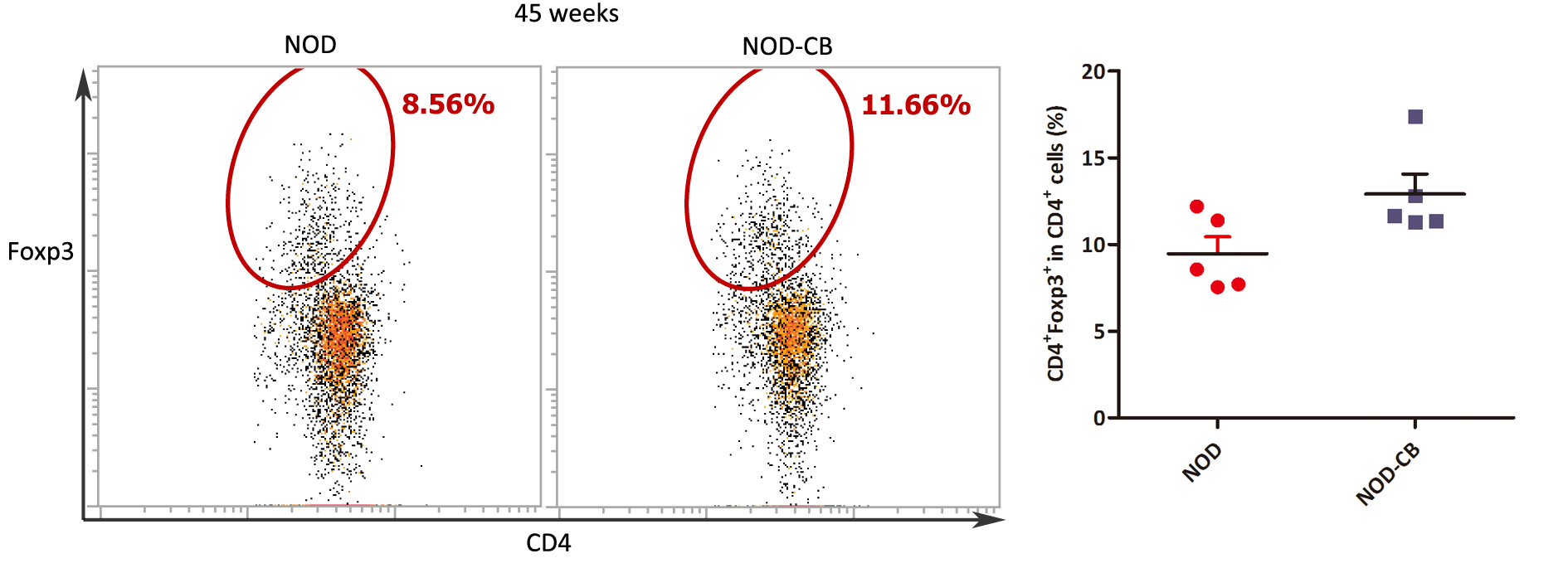

Supplement: Figure S4 — Percent CD4+Foxp3+ cells in pancreas as indicated at 45 weeks of age. [file image_4.tif]

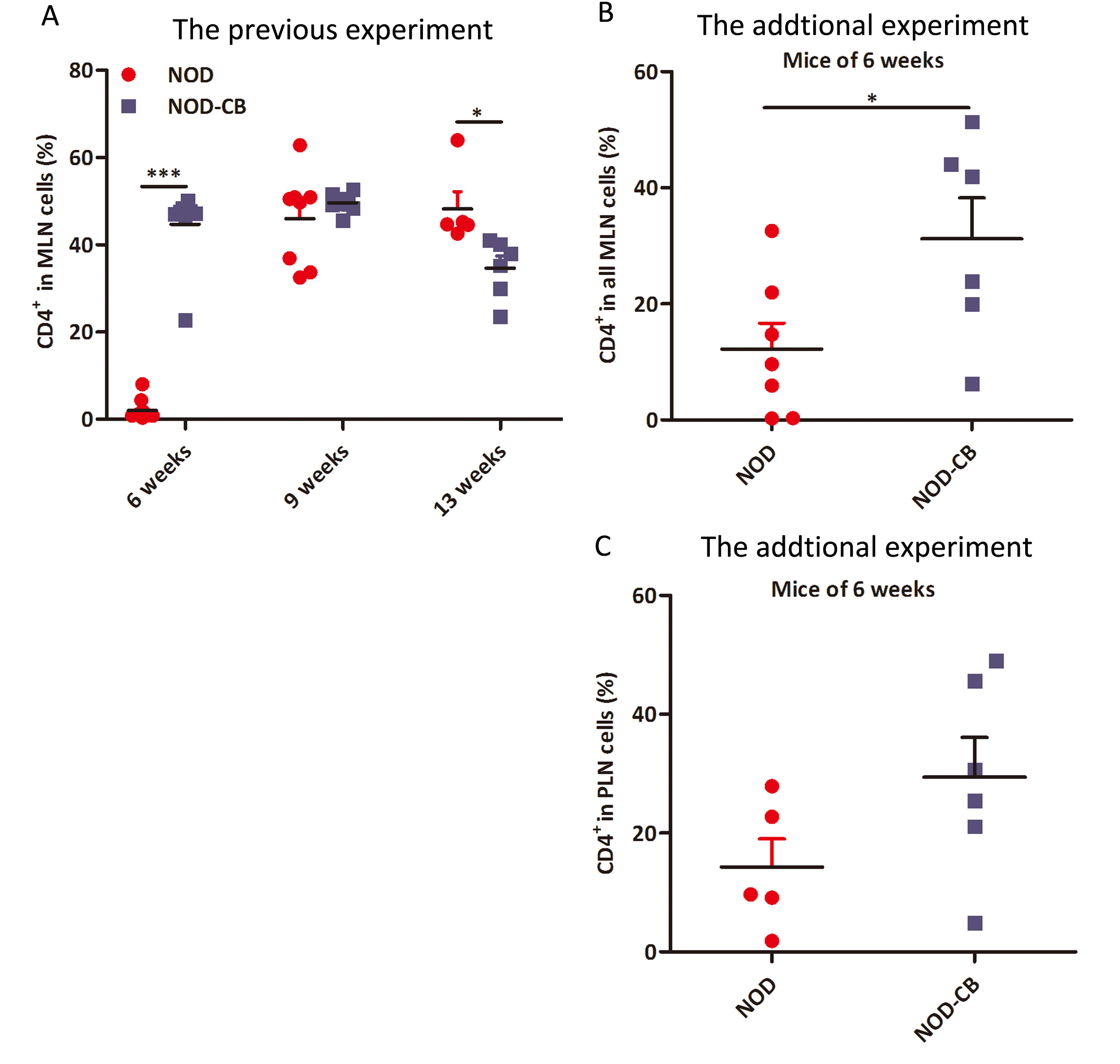

Supplement: Figure S5 — Percent CD4+ cells in MLN and PLN. (A) Percent CD4+ cells in MLN as indicated at 6, 9, and 13 weeks of age in the previous experiments using the antibody of CD4 (eBioscience, FITC). (B) Percent CD4+ cells in MLN e as indicated at 6 weeks of age in the additional experiments using the new antibody of CD4 (Miltenyi, PE-vio770). (C) Percent CD4+ cells in PLN as indicated at 6 weeks of age in the additional experiments using the antibody of CD4 (Miltenyi, PE-vio770). [file image_5.tif]

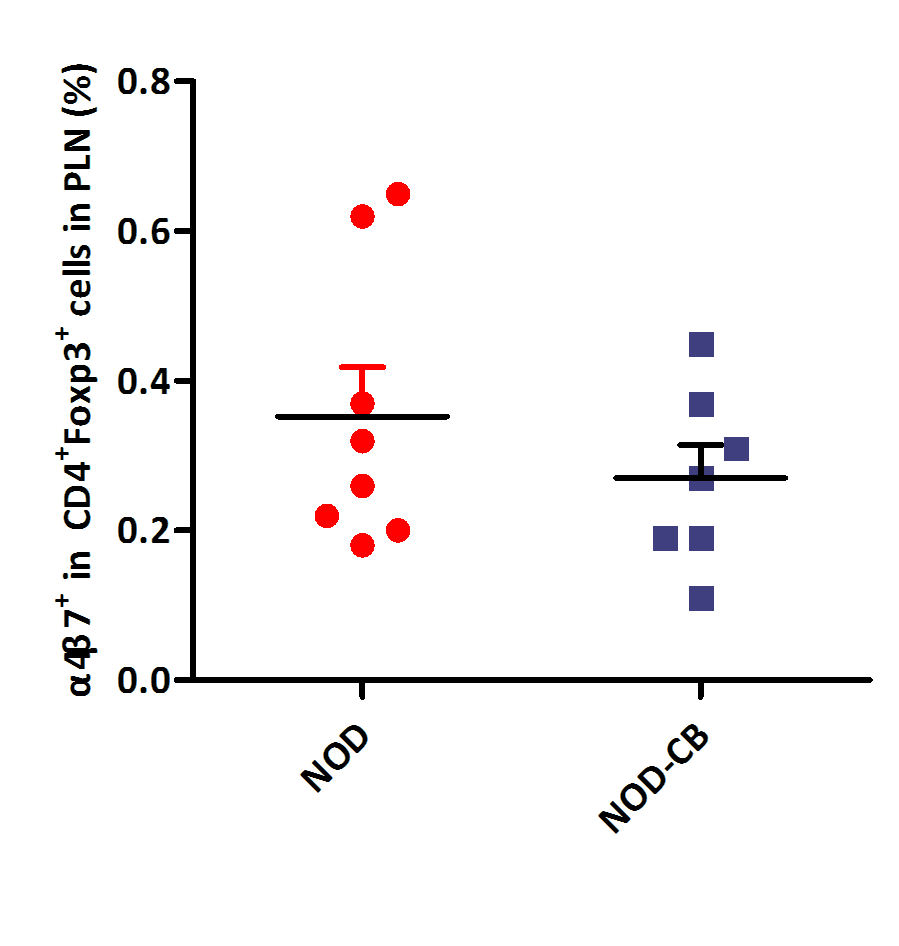

Supplement: Figure S6 — Percent α4β7+ cells in PLN Tregs. Data are mean ± SEM (n = 5–8 mice per group). *, **, *** p < 0.05, p < 0.01, p < 0.001 vs NOD control mice by t-test. [file image_6.tif]

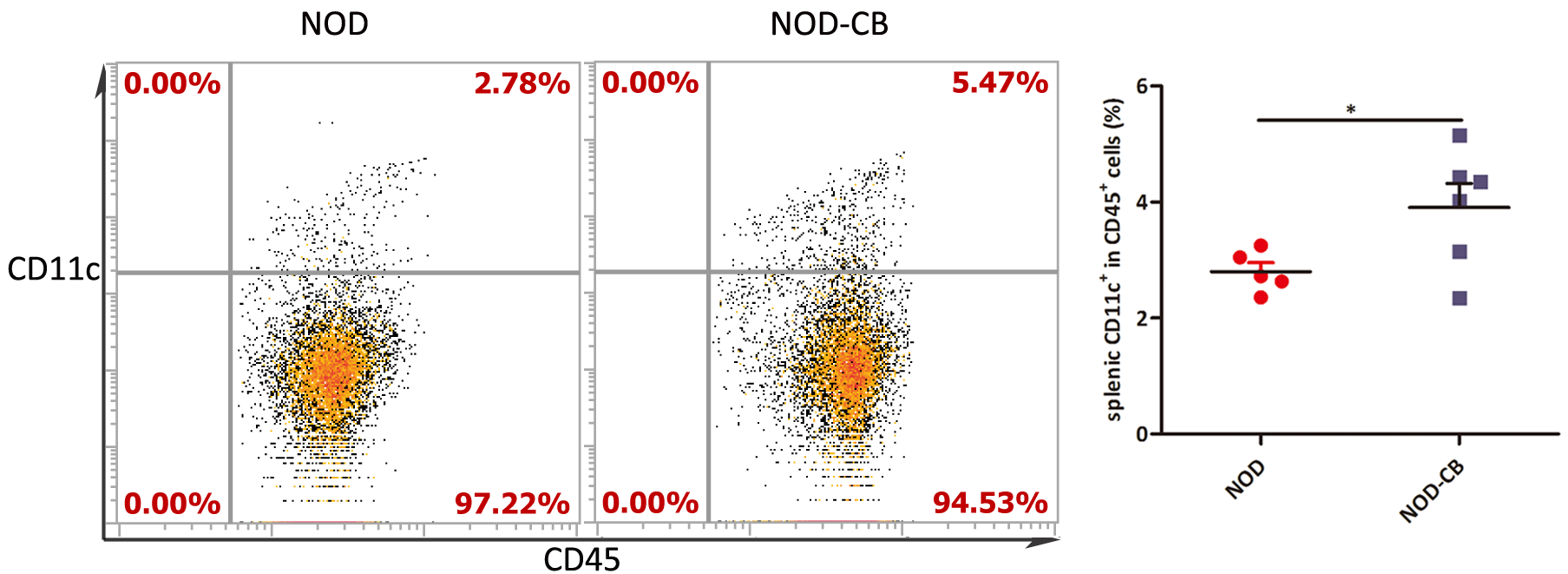

Supplement: Figure S7 — Percent CD11c+ cells in splenic CD45+ cells. *, **, *** p < 0.05, p < 0.01, p < 0.001 vs NOD control mice by t-test. [file image_7.tif]
