# Supplementary material for: Clostridium butyricum CGMCC0313.1 Protects against Autoimmune Diabetes by Modulating Intestinal Immune Homeostasis and Inducing Pancreatic Regulatory T Cells
Source: Front Immunol. 2017 Oct 19;8:1345. doi: 10.3389/fimmu.2017.01345 (PMC5654235; doi:10.3389/fimmu.2017.01345)
Supplement: Table S1 — Information of antibody for FACS. [file table_1.doc]

**Supplementary Table S1- Information of antibody for FACS**

| Antibody | Brand | Fluorescence |
| --- | --- | --- |
| CD4 | eBioscience | FITC |
| CD25 | eBioscience | Apc |
| Fxop3 | eBioscience | PE |
| CD11c | BioLegend | Brilliant Violet 421 |
| IFN-γ | eBioscience | PE |
| IL-4 | eBioscience | APC |
| IL-17A | BioLegend | Brilliant Violet 421 |
| Rat IgG2a K Isotype Control | eBioscience | PE |
| Rat IgG1 K Isotype Control | eBioscience | PE |
| Rat IgG1 K Isotype Control | eBioscience | APC |
