# Supplementary material for: Clostridium butyricum CGMCC0313.1 Protects against Autoimmune Diabetes by Modulating Intestinal Immune Homeostasis and Inducing Pancreatic Regulatory T Cells
Source: Front Immunol. 2017 Oct 19;8:1345. doi: 10.3389/fimmu.2017.01345 (PMC5654235; doi:10.3389/fimmu.2017.01345)
Supplement: Table S2 — Primer sequences used in this study for microbial abundance. [file table_2.doc]

**Supplementary Table S2-**Primer sequences used in this study for microbial abundance

| Gene name | Gene symbol | Sequence |
| --- | --- | --- |
| *butyrate kinase gene* | *buk* | Buk-5F1 CCATGCATTAAATCAAAAAGC  Buk-5F2 CCATGCGTTAAACCAAAAAGC  Buk-6R1 AGTACCTCCACCCATGTG  Buk-6R2 AATACCTCCGCCCATATG  Buk-6R3 AATACCGCCRCCCATATG |
| Total bacteria | Total | Forward5’- GCAGGCCTAACACATGCAAGTC  Reverse 5’-CTGCTGCCTCCCGTAGGAGT |
| *butyryl-coenzym* | *but-CoA* | Forward5’-GCIGAICATTTCACITGGAAYWSITGGCAYATG  Reverse 5’-CCTGCCTTTGCAATRTCIACRAANGC |
| Clostridium butyrate CGMCC0313.1 | CB0313*.1* | Forward5’-CCTCCTTTCTATGGAGAAATCTAGCA  Reverse 5’-TGTAGCTTGACCTTTTTAAGTTTTGA |
| Clostridial (cluster XIVa) | XIVa | Forward5’- CGGTACCTGACTAAGAAGC  Reverse 5’-AGTTTYATTCTTGCGAACG |
| Clostridial (cluster IV) | IV | Forward5’-TTA CTG GGT GTA AAG GG  Reverse 5’-TAG AGT GCT CTT GCG TA |
