# Supplementary material for: Clostridium butyricum CGMCC0313.1 Protects against Autoimmune Diabetes by Modulating Intestinal Immune Homeostasis and Inducing Pancreatic Regulatory T Cells
Source: Front Immunol. 2017 Oct 19;8:1345. doi: 10.3389/fimmu.2017.01345 (PMC5654235; doi:10.3389/fimmu.2017.01345)
Supplement: Table S3 — Information of antibody for FACS for the experiment of regulatory T cells migration. [file table_3.doc]

**Supplementary Table S3- Information of antibody for FACS for the experiment of Tregs migration**

| Antibody | Brand | Fluorescence |
| --- | --- | --- |
| CD4 | miltenyi | PE-vio770 |
| CD25 | eBioscience | Alexa Fluor 488 |
| Fxop3 | eBioscience | APC |
| Integrin α4β7 | BioLegend | PE |
