# Supplementary material for: Clostridium butyricum CGMCC0313.1 Protects against Autoimmune Diabetes by Modulating Intestinal Immune Homeostasis and Inducing Pancreatic Regulatory T Cells
Source: Front Immunol. 2017 Oct 19;8:1345. doi: 10.3389/fimmu.2017.01345 (PMC5654235; doi:10.3389/fimmu.2017.01345)
Supplement: Table S4 — Information of antibody for FACS for the experiments of CD45+CD11c+. [file data_sheet_4.doc]

**Supplementary Table S4- Information of antibody for FACS for the experiments of CD45^+^CD11c^+^.**

| Antibody | Brand | Fluorescence |
| --- | --- | --- |
| CD45 | miltenyi | PE-vio-770 |
| CD11c | BioLegend | Alexa Fluor 647 |
